# Single-cell transcriptomics revealed molecular vulnerability in a human midbrain-like organoid model of Parkinson’s disease

**DOI:** 10.1016/j.isci.2026.114674

**Published:** 2026-01-10

**Authors:** Jessica Jiaxin Xie, Matas Vitkauskas, Quyen Do, Tzuen Yih Saw, Alfred Xuyang Sun, Lin Yang, Tuck Wah Soong, Kah-Leong Lim, Eng-King Tan, Huck-Hui Ng, Jinyue Liu

**Affiliations:** 1 Genome Institute of Singapore, Agency for Science, Technology and Research, Singapore 138672, Singapore; 2 Department of Neurology, National Neuroscience Institute (Singapore General Hospital Campus), Singapore 308433, Singapore; 3 Duke-NUS Medical School, National University of Singapore, Singapore 169857, Singapore; 4 Department of Biochemistry, Yong Loo Lin School of Medicine, National University of Singapore, Singapore 117597, Singapore; 5 Department of Biological Sciences, National University of Singapore, Singapore 117558, Singapore; 6 Lee Kong Chian School of Medicine, Nanyang Technological University, Singapore 308232, Singapore; 7 School of Biological Sciences, Nanyang Technological University, Singapore 637551, Singapore

**Keywords:** Disease, Molecular neuroscience, Integrative aspects of cell biology, Transcriptomics

## Abstract

The human midbrain-like organoid (hMLO) is a key model system for investigating pathological features of Parkinson’s disease (PD), yet how its molecular landscape relates to cellular vulnerability in PD remains unclear. We performed in-depth single-cell characterization of our previously established hMLO model up to 150 days *in vitro*. Our hMLOs exhibited physiological cell types and broad topographical patterning, consistent with features of the human fetal midbrain. We further identified four distinct dopamine-producing neurons (DaN) subtypes whose molecular profiles span a key transcriptomic axis in the selective vulnerability of DaNs in PD. Knockout of *PARK7*, a highly penetrant PD-causing gene, in hMLOs induced cell type-dependent molecular perturbations in mitochondrial activity and synapse biology, and recapitulated PD pathophysiology, including α-synuclein aggregation, Lewy Body-like inclusions, and DaN degeneration with extended culture. This study highlights the utility of our hMLO model in manifesting pathological features and cell type-specific vulnerability, enabling mechanistic studies into PD pathophysiology.

## Highlights

• Comprehensive multi-omics evaluation of human midbrain-like organoids
• Identified organoid analogs of human midbrain dopaminergic subtypes
• Cell identities were differentially enriched for subsets of PD-linked genes
• PARK7 loss induced broad mitochondrial defects but restricted synaptic dysfunction

## Graphical abstract

**Figure:**
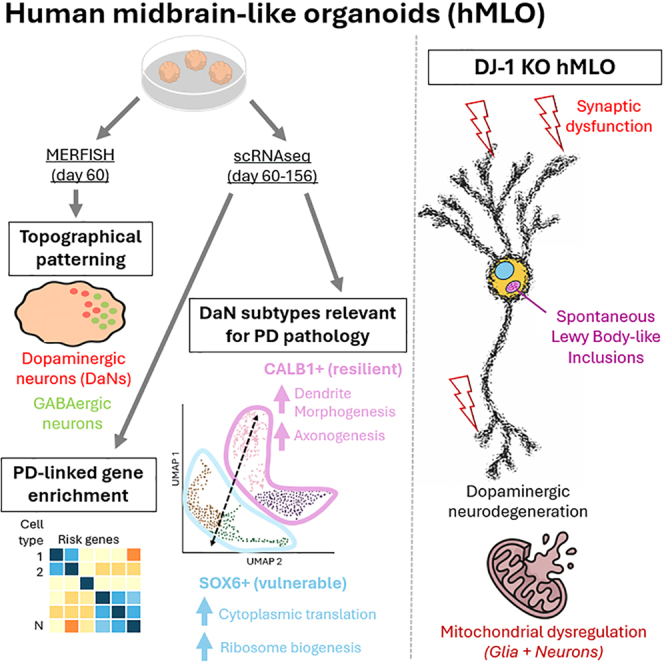

## Introduction

Parkinson’s disease (PD) is characterized by the selective degeneration of dopamine-producing neurons (DaNs) in the substantia nigra pars compacta (SNpc) of the midbrain.^1^ Its pathogenesis and progression have long been recognized as multi-factorial and multi-stage, differentially affecting a broad spectrum of cell types, in addition to DaNs, within the affected regions. Various *in vitro* methods have highlighted the necessity of complex culture systems capable of recapitulating both the intrinsic biology, cellular diversity, and tissue microenvironment to better facilitate pathological manifestations for mechanistic studies.^2^^,^^3^^,^^4^^,^^5^ Among these, human midbrain-like organoids (hMLOs) have emerged as a highly promising experimental system.^6^^,^^7^ Their three-dimensional structure offers a more faithful representation of intrinsic cell diversity and architecture, providing a more accurate simulation of the molecular features and cell-cell interactions critical to understanding PD pathology. Early evidence has demonstrated the complex self-organization and cellular diversity within hMLOs reminiscent of the human midbrain.^6^ hMLO models harboring major risk factors of PD could spontaneously generate many key aspects of PD pathology, including the loss of DaNs. The formation of Lewy Body-like inclusions,^7^^,^^8^ a phenomenon which is challenging to establish in the traditional two-dimensional adherent cell culture, has also been recapitulated.

Recent advances in single-cell sequencing have offered a more detailed and granular perspective of hMLO early development and cellular composition.^9^ Single-cell profiling of organoid models of PD has also uncovered potentially early developmental changes in PD.^5^^,^^10^ However, it remains unclear how hMLO molecular landscape evolves over development, and whether these transcriptomic profiles are predictive of cell type-specific vulnerability or resilience observed clinically.

In this study, we have conducted a comprehensive single-cell and spatial characterization of our pioneering hMLO system,^6^^,^^7^ across a range of ages and into the later stages of organoid maturation. We demonstrated that our hMLOs recapitulate key midbrain cellular identities and DaN subtypes that are stratified along a major transcriptomic axis in the selective vulnerability of DaNs in PD. Many of these cell identities exhibit cell type-specific PD risk enrichment profiles. We extended our hMLO system to PD modeling by characterizing molecular perturbations following knockout of *PARK7*, an autosomal recessive PD-causing gene that encodes the DJ-1 protein. Broad upregulation of mitochondria-related activities across multiple cell types, but DaN-specific downregulation of synaptic biology accompanied key pathological hallmarks of PD, such as *de novo* formation of α-synuclein-positive Lewy body-like inclusions (LBLIs) and reduced expression of DaN markers in long-term culture. Overall, the study not only enhances the utility of our hMLO model for understanding PD pathogenesis but also lends insights into the molecular correlates of differential cellular vulnerabilities in PD.

## Results

### hMLOs exhibit physiologically relevant cell type diversity and regional identity

**Figure 1:**
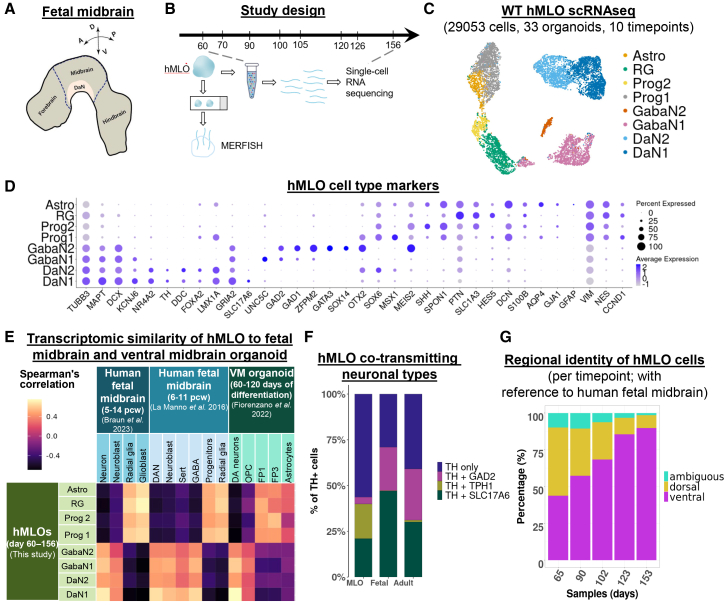
hMLOs exhibit physiologically relevant cell type diversity and regional identity (A) Schematic of the human fetal midbrain. (B) Study design timeline. Single-cell RNA sequencing (scRNAseq) was performed across the period of day 60–156 (Table S1). MERFISH was performed at day 60 (Table S2). (C) UMAP scatterplot of 29053 scRNAseq transcriptomic profiles of day 60–153 wildtype (WT) hMLO cells (*n* = 33 hMLOs, 10 timepoints), colored by cell type. Astro - astrocytes, RG - radial glia, Prog2 – progenitors 2, Prog1 – progenitors 1, GabaN2 – GABAergic neurons 2, GabaN1 – GABAergic neurons 1, DaN2 – dopaminergic neurons 2, DaN1 – dopaminergic neurons 1. (D) Dot plot shows the expression of cell type markers (log-normalized, scaled by 10,000, and centered). (E) Heatmap shows Spearman’s correlation of transcriptomic profiles of hMLO cell types with those from human ventral midbrain (VM) organoid^9^ and human fetal midbrain.^11^^,^^12^ The correlation used 2015 genes that are highly variable across all samples. DA neurons – dopaminergic neurons, Sert – serotonergic neurons, GABA – GABAergic neurons, OPC – oligodendrocyte precursor cells, and FP – floorplate cells. (F) Proportions of DaNs that express TH + alone, or that co-express GAD2, TPH1, or SLC17A6 in hMLOs, human fetal midbrain, and human adult midbrain. (G) Stacked barplot indicates the proportions of each regional identity in hMLOs at different time points. Regional labels are with reference to the human fetal midbrain.

To evaluate the cellular composition of our previously established hMLO system^6^ (Figure 1A), we performed time-course single-cell RNA sequencing (scRNAseq) on 33 hMLOs derived from wild-type (WT) H9 human embryonic stem cells (hESCs), across days 60–156 (Figure 1B and Table S1, *n* = 3 organoids per timepoint). A total of 29053 cells passed quality checks (Figure S1A, STAR Methods). Louvain clustering revealed eight transcriptomically distinct cell populations (Figures 1C and 1D), all of which were represented at each timepoint (Figure S1B).

We identified two progenitor clusters, progenitors 1 (Prog1) and 2 (Prog2), that expressed neurodevelopmental genes such as *VIM, DCN,* and *SPON1*^13^ as well as key midbrain markers *FOXA2*^14^*, TFF3*,^15^ and *SOX6*.^16^ However, only Prog1, and not Prog2, expressed other midbrain markers such as *OTX2*,^17^ as well as *LMX1A* and its downstream target *MSX1*.^18^ Radial glia (RG) marked by *VIM*^19^*, SLC1A3*^20^*, HES5,*^21^ and *SOX6,*^22^ and a small cluster of astrocytes (Astro) that expressed astrocyte-committed genes *GFAP*^23^*, AQP4*^24^*, S100A10,*^25^ and *PTGDS*^26^ were also identified (Figure 1D).

We detected four neuronal clusters that were enriched in *DCX, TUBB3,* and *MAPT* (Figure 1D). 37.6% of all neurons were midbrain DaNs expressing dopamine synthesis enzymes *TH* and *DDC*, and DaN fate specification markers *NR4A2, EN1, PBX1,* and *PBX3*.^27^^,^^28^ DaN1 had greater expression of postmitotic DaN markers *TH, TUBB3, MAPT,* and *KCNJ6,* as well as reduced expression of DaN transcription factors *SOX6, OTX2,* and *MSX1* compared to DaN2. 23.4% of all neurons were GABAergic neurons (GabaN) defined by expression of GAD1/2, the key enzymes catalyzing GABA synthesis.^29^ GABAergic neurons 2 (GabaN2) expressed dorsal midbrain markers *GATA3, ZFPM2, SOX14, OTX2,* and *MEIS2*^30^^,^^31^^,^^32^ while GABAergic neurons 1 (GabaN1) exhibited features of neuronal function including *UNC5C,*^33^ *EPHA5,*^34^ and *KCNJ6*.

To evaluate the identity of cluster labels from our hMLOs, we compared the transcriptomic profiles of our hMLO cell types against published single-cell atlases of human fetal midbrain^11^^,^^12^ and an existing ventral midbrain organoid model.^9^ Spearman’s correlation analysis indicated positive correlation of our hMLO neuronal types with corresponding neuronal classes in the references (*r =* 0.17 to 0.71), but not non-neuronal cell types (*r =* −0.69 to −0.42) (Figure 1E). Conversely, the four non-neuronal clusters in our hMLOs were negatively correlated with reference neuronal classes (r = −0.96 to −0.35) but positively correlated with non-neuronal counterparts (r = 0.54 to 0.73) (Figure 1E). Our hMLO system thus broadly recapitulates the molecular identity of key cell types within the human fetal midbrain.

Neurotransmitter phenotype is widely recognized as a useful classifier for neuronal identity.^35^ Midbrain dopaminergic neurons are known to co-release GABA,^36^ serotonin,^37^ or glutamate,^38^ marked by co-expression of GAD2, TPH1, and SLC17A6, respectively. To find out if these co-transmitting populations are present in our hMLOs, we quantified the proportions of TH + neurons that co-expressed GAD2, TPH1, or SLC17A6. Among hMLO DaNs, 56.4% expressed TH solely, while 3.68%, 19%, and 21% co-expressed GAD2, TPH1, and SLC17A6, respectively. As a reference, we examined TH + cells in publicly available datasets from human fetal and adult midbrain and found similar co-transmitting subtypes (Fetal^12^: 29.0% TH + only, 23.8% TH+/GAD2+, 0.2% TH+/TPH1+, and 47.0% TH+/SLC17A6+; Adult^39^: 40.9% TH + only, 28.0% TH+/GAD2+, 1.01% TH+/TPH1+, and 30.1% TH+/SLC17A6+) (Figure 1F). Notably, we detected a small population (3.68%) of TH+/GAD2+ neurons (Figure 1F), consistent with a co-transmitting population recently reported in the human midbrain atlas.^39^ Immunocytochemistry confirmed dual positivity of TH and GABA (Figure S1C). This analysis demonstrates that our hMLO protocol was able to generate physiologically relevant diversity in co-transmitting neuronal types.

We further explored whether hMLOs preferentially model certain midbrain subregions. We focused on the dorsoventral axis of the fetal midbrain as the majority of midbrain dopaminergic neurons (including those in the substantia nigra and VTA) originate from the ventral midbrain. We first identified 207 fetal midbrain regional markers (109 ventral, 98 dorsal) from the human fetal midbrain atlas.^12^ Among the ventral markers were floorplate markers *FOXA2* and *FOXA1,*^40^ whose expression is indicative of the substantia nigra pars compacta (SNpc) lineage. Dorsal markers included *PAX7, PAX3,* and *MEIS2*^41^ (Figure S1D). Using these markers, we scored the regional identity of individual hMLO cells and found that the proportion of cells with ventral identity increased from 43.8% at day 65–89.7% at day 153 (tau = 1, *p* = 0.027, Mann-Kendall test), while the proportion of cells with dorsal identity decreased from 46.4% at day 65 to 8.7% at day 153 (tau = −1, *p* = 0.027, Mann-Kendall test) (Figures 1G and S1E). There were also cells whose transcriptomic profiles lacked distinct dorsal or ventral midbrain signatures. The proportion of such ambiguous cells decreased from 9.8% at day 65 to 1.5% at day 153 (tau = −0.8, *p* = 0.086, Mann-Kendall test) (Figure 1G), suggesting the progressive regionalization of hMLOs. Notably, the majority of GabaN1 and GabaN2 are of dorsal origin (64.7% and 88.0%, respectively), making up 60.0% of all dorsal cells (Figures S1F and S1G). These results suggest that after the initial patterning phase, hMLOs continue to undergo dynamic regional specification toward the ventral midbrain with age.

### Spatial transcriptomics reveal tissue organization of hMLOs

Given that our hMLOs displayed a mix of dorsoventral identities, we next explored the spatial organization of cell types within our hMLOs using multiplexed error-robust fluorescence *in situ* hybridization (MERFISH).^42^^,^^43^ We analyzed the spatial distribution of 136 genes in 10 sections from three-day 60 hMLOs (Table S2). These genes included those that mark all neurons (*MAPT* and *DCX*^44^^,^^45^), Astro (*AQP4*^24^), RG (*SLC1A3*^20^), and GabaNs (*GAD1*) (Figures S2A–S2C). Since the *TH* transcript sequence contains too few unique regions that can be targeted by the minimum probe set required for MERFISH detection, we used co-expression of *NR4A2, LMX1A,* and *MAPT* instead to identify DaNs. Genes involved in hypoxia, metabolism, lysosomal function, and dopamine metabolism were also included. Day 60 was selected as hMLOs carrying mutations in PD-associated genes have been shown to exhibit PD-like pathology at day 60.^7^

**Figure 2:**
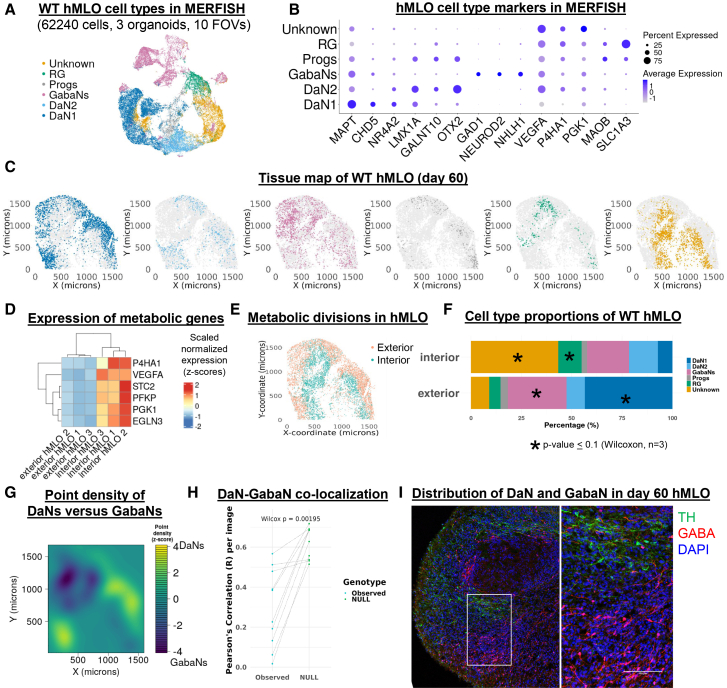
Spatial transcriptomics reveal tissue organization of hMLOs (A) UMAP embedding of clustered MERFISH gene-cell matrix (136 genes × 62240 cells). RG – radial glia, Progs – progenitors, GabaNs – GABAergic neurons, DaN2 – dopaminergic neurons 2, and DaN1 – dopaminergic neurons 1. (B) Dot plot shows expression cell type markers in MERFISH data (log-normalized, scaled by 10,000, and centered values). (C) Tissue maps of cell centroids corresponding to different cell type identities in a WT hMLO sample. (D) Heatmap of scaled log-normalized expression of selected metabolic genes in each hMLO interior and exterior. (E) Tissue map of a WT hMLO sample shows cells marked as interior or exterior based on their binarized metabolic index. (F) Barplot shows mean cell type proportions across all WT samples in hMLO interior or exterior. Asterisks indicate cell types for which the average percentages of cells that are in the hMLO interior were significantly different from those in the exterior (Wilcoxon ranked sum test, see Figure S3E). (G) Contour plot of point density difference between DaNs (DaN 1 + DaN 2) and GabaNs in a WT hMLO. (H) Observed Pearson’s correlation values of binned cell centroids of DaNs and GabaNs and corresponding median Pearson’s correlation values obtained from permuting the centroids 10,000 times (null distribution). Lines connect matched images. *p* = 0.00195, paired Wilcoxon ranked sum test. (I) Immunostaining of TH (green), GABA (red), and DAPI (blue) in an independent day 60 hMLO. Scale: 100 μm.

We identified five cell types corresponding to those in our scRNAseq data (Figures 2A and 2B) and mapped their spatial distributions (Figures 2C and S2D). We noticed that some cell types were visibly enriched in certain parts of the hMLOs and hypothesized that their proportions might have been impacted by the intrinsic metabolic gradient that is known to occur in organoids.^46^^,^^47^^,^^48^ To ask how the various cell types respond to metabolic stress, we first established a metabolic index based on the expression of glycolysis and hypoxia-related genes, namely *P4HA1, STC2, PGK1, VEGFA, EGLN3,* and *PFKP* (Figure 2D). Their differential expression largely correlated with the distance from the organoid surface and was consistently observed across all samples and cell types (Figures S3A and S3B). Binarizing the cells based on their metabolic index (Figure S3C) revealed a more hypoxic interior and a less hypoxic exterior (Figures 2D, 2E, and S3D), reflecting the core of hypoxic stress commonly seen within organoids.^46^^,^^47^^,^^49^ All cell types were represented in the hMLO interior and exterior (Figure 2F). However, the proportion of DaN1, but not that of the less mature DaN2, was higher in the hMLO exterior (*p = 0.1*, Wilcoxon ranked-sum test), reflecting the greater metabolic demand of more mature neurons (Figures 2F and S3E). Certain cells (labeled “Unknown”) solely exhibited metabolic genes and were enriched in the hMLO interior (*p =* 0.1, Wilcoxon ranked-sum test) (Figures 2F and S3E), suggesting that their cellular identities might have been compromised by hypoxia. Indeed, we observed interior localization of CC3+ necrotic cells relative to VIM+ proliferative cells and MAP2+ neuronal cells by immunostaining (Figure S3F). CC3+ cells did not colocalize with VIM or MAP2, consistent with their “unknown” label.

We used GabaNs and DaNs as a proxy for the dorsal and ventral midbrain, respectively (Figures S1F and S1G) and compared the spatial cell distribution of GabaNs to that of DaNs. We correlated binned cell abundances of DaNs to those of GabaNs and confirmed that their relative distributions - irrespective of their relative orientation within the hMLO - were non-random in 9 out of 10 images (*p <* 0.05, one-tailed permutation test) (Figures 2G, S3G, and S3H). The observed correlation values were significantly lower than those obtained using randomized cell abundances (*p* = 0.00195, paired Wilcoxon ranked sum test) (Figure 2H). Immunohistochemistry corroborated the spatial separation of the two cell types at the same timepoint (Figures 2I and S3I). Together, the lack of co-localization of DaNs and GabaNs suggests a broad topographical patterning early during hMLO development.

### hMLO cell types and DaN subtypes vary in PD vulnerability

**Figure 3:**
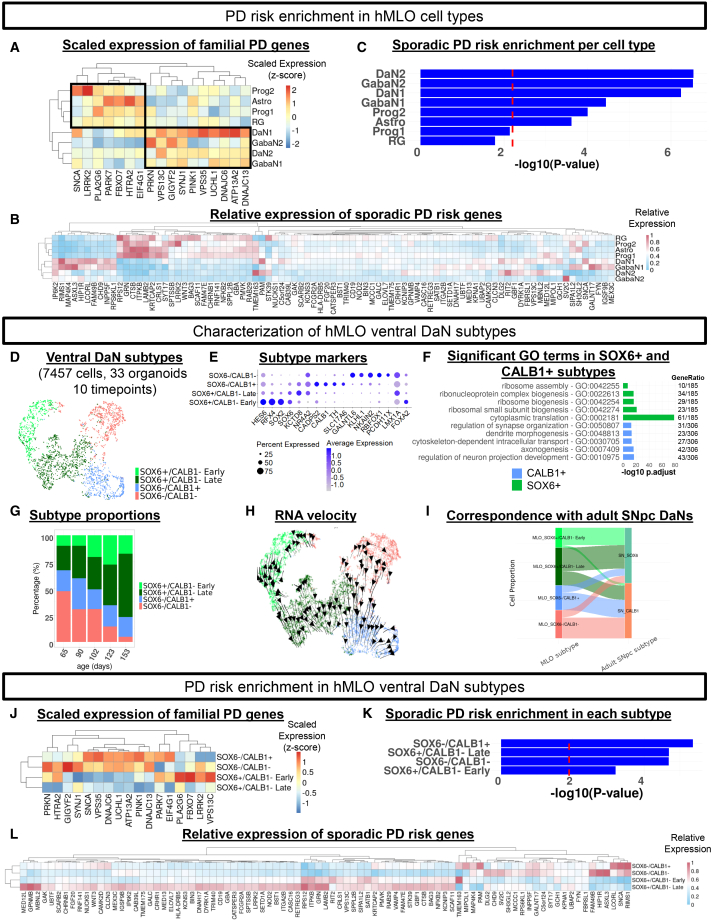
hMLO cell types and DaN subtypes vary in PD vulnerability (A) Heatmap of scaled log-normalized expression of familial PD (fPD) genes per cell type (z-scores, scaled by column). (B) Heatmap shows the relative expression of 87 sporadic PD (sPD) genes within each cell type. (C) Barplot shows the enrichment of sPD risk variants in hMLO cell types. Dotted line indicates Bonferroni-corrected p_adj<0.05 (Fisher’s exact test). (D) UMAP scatterplot of hMLO DaN subtypes along the SOX6-CALB1 expression axis. (E) Dot plot shows the expression of hMLO DaN subtype markers (log-normalized, scaled by 10,000, and centered). (F) Barplot of the top 5 GO terms in CALB1+ and SOX6+ populations. (G) Stacked barplot shows SOX6/CALB1 subtype proportions by hMLO age. (H) RNA velocities projected onto UMAP-based embedding of hMLO DaN subtypes. (I) Alluvial plot shows correspondence between hMLO DaN subtypes and DaN subtypes within adult substantia nigra pars compacta (SNpc).^16^ (J) Heatmap of scaled log-normalized expression of familial PD (fPD) genes in hMLO DaN subtypes (z-scores, scaled by column). (K) Barplot shows the enrichment of sPD risk variants in hMLO DaN subtypes. Dotted line indicates Bonferroni-corrected p_adj<0.05 (Fisher’s exact test). (L) Heatmap shows the relative expression of 87 sporadic PD (sPD) genes within DaN subtypes.

Having established the physiological relevance of our hMLOs, we next investigated their utility for modeling PD. We examined the expression of 17 genes linked to rare familial forms of PD (fPD)^50^ in our scRNAseq data. All 17 genes were expressed in all eight hMLO cell types (Figure 3A). Hierarchical clustering of these genes across all cell types revealed two major clusters, one comprising neuronal identities (*PRKN, VSP13C, SYNJ1, PINK1, VSP35, UCHL1, DNAJC6, ATP13A2, DNAJC13*) and another comprising non-neuronal identities (*SNCA, LRRK2, PLA2G6, PARK7, FBXO7, HTRA2, EIF4G1*) (Figure 3A).

We next curated a list of 93 unique risk genes associated with small nucleotide polymorphisms (SNPs) identified from a meta-genome wide association study (GWAS) for sporadic PD (sPD) in the European^51^ and Asian^52^ populations. Between 88 and 93 of these sPD risk genes were expressed in various hMLO cell types. Similar to fPD genes, several sPD risk genes were enriched in either neuronal (*RIMS1, MAP4K4, IP6K2, RIT, DLG2*) or progenitor/astrocyte clusters (*RAB29, CTSB, ITPKB, LAMB2, RPS12,* and *GRN*) (Figure 3B). Thus, different risk variants may be implicated at different stages of development and in different cell types. We further performed risk enrichment analysis for the 9 to 18 sPD genes that exceeded a baseline expression threshold (average count per cell (for each cell type) > 1) (Table S3). They were particularly enriched in neuronal cell types, such as GabaN2 (*p* = 3.30e-7), GabaN1 (*p* = 2.64e-6), DaN2 (*p* = 7.12e-6), and DaN1 (*p* = 1.06e-5) (Figure 3C). This result concurs with previous findings implicating increased sPD risk among neuronal cell types, particularly DaNs.^16^^,^^53^

Since different DaN subtypes in the ventral midbrain are differentially vulnerable in PD,^16^ we asked if our hMLO ventral DaNs could be further subtyped. We found four populations stratified along the SOX6–CALB1 axis, which is characteristic of DaN ontogenic histories and selective vulnerability^12^^,^^16^^,^^39^^,^^54^ (Figures 3D and S4A–S4D). The two SOX6+/CALB1- populations displayed high expression of SOX6 in conjunction with low CALB1, suggestive of the vulnerable DaN population in the SNpc.^11^^,^^16^^,^^54^ One population was enriched for early progenitor markers *HES6, RFX4,* and *SOX2* (hereby termed SOX6+/CALB1- Early) while the other was enriched for the mature DaN marker *NR4A2* (hereby termed SOX6+/CALB1- Late) (Figure 3E). On the other hand, the SOX6-/CALB1+ population expressed *SLC17A6*, which was found to be associated with resilient DaNs in the ventral midbrain^54^^,^^55^^,^^56^ (Figure 3E).

We performed a differential gene expression analysis between the two SOX6+/CALB1-subtypes and the SOX6-/CALB1+ subtype to elucidate the molecular differences that may underlie their reported differential susceptibility in PD. GO analysis showed upregulation of genes associated with cytoplasmic translation and ribosomal functions in the combined SOX6+ subtypes. In contrast, the SOX6-/CALB1+ subtype was enriched in GO terms associated with neuron projection and dendrite development (Figure 3F).

Proportions of ventral DaN subtypes in hMLOs evolved over time (Figure 3G). The SOX6+/CALB1- Early population remained relatively stable at ∼17% across all timepoints (tau = 0.6, *p* = 0.22, Mann-Kendall test) while the SOX6+/CALB1- Late population increased from 23% at day 65 to 59% at day 153 (tau = 1, *p* = 0.027, Mann-Kendall test) (Figure 3G). The frequency of SOX6-/CALB1+ cells remained consistent at ∼21% (tau = −0.4, *p* = 0.46, Mann-Kendall test) (Figure 3G). We also observed a SOX6-/CALB1- population expressing early midbrain developmental genes such as *RBFOX1* and *KLHL1*^57^ (Figure 3E). Its proportion decreased substantially from 47.5% at day 65 to 4.8% at day 153 (tau = −0.8, *p* = 0.086, Mann-Kendall test) (Figure 3G). These results suggest that there is a gradient of maturation among ventral DaNs during hMLO development.

To test this hypothesis, we performed RNA velocity on all hMLO ventral DaNs. Two main trajectories were observed, one extending from the SOX6+/CALB1- Early to SOX6+/CALB1- Late population, and the other from the SOX6-/CALB1- to SOX6-/CALB1+ population (Figure 3H). These trajectories were consistent with the molecular profiles and underlying gene expression dynamics of each subtype (Figures 3E, S4E, and S4F), as well as the later emergence of CALB1 expression *in vivo.*^58^ SOX6-/CALB1- subtype also shared early markers of the SOX6-/CALB1+ population *in vivo* (*FSTL4, EPHB1,* and *FXYD6*^54^) (Figure S4D), further supporting the developmental continuum observed.

To find out if the hMLO SOX6-CALB1 transcriptional axis correlates with that in the human adult midbrain, we integrated our hMLO ventral DaNs with adult midbrain DaNs^16^ (Figure S4G). 62.3% of hMLO SOX6+/CALB1- Late neurons and 87.4% of SOX6+/CALB1- Early neurons aligned with the adult *SOX6*^*+*^ subtype, while 71.5% of the SOX6-/CALB1+ population matched the adult *CALB1+* subtypes (Figure 3I). Notably, 73.7% of the SOX6-/CALB1- population also corresponded to the adult *CALB1+* subtypes, consistent with our RNA velocity findings. Thus, hMLO DaN subtypes stratified along the SOX6-CALB1 axis are reminiscent of their *in vivo* adult counterparts.

Following the subtyping of our hMLO ventral DaNs, we conducted a risk enrichment analysis of fPD genes and sPD risk loci in the four ventral DaN subtypes and found differential enrichment along the SOX6-CALB1 axis (Figures 3J–3L). All four subtypes also showed significant cumulative sPD risk (SOX6-/CALB1-: *p = 2.07e-5*; SOX6-/CALB1+: *p* = 4.33e-6, SOX6+/CALB1- Early: *p* = 6.26e-4, SOX6+/CALB1-Late: *p* = 2.02e-5) (Figure 3K). These findings align with previous reports of selective neuronal vulnerability along the SOX6-CALB1 axis in PD,^16^ supporting the use of organoid models in PD.

### An isogenic PARK7 knockout revealed cell type-dependent molecular perturbations

**Figure 4:**
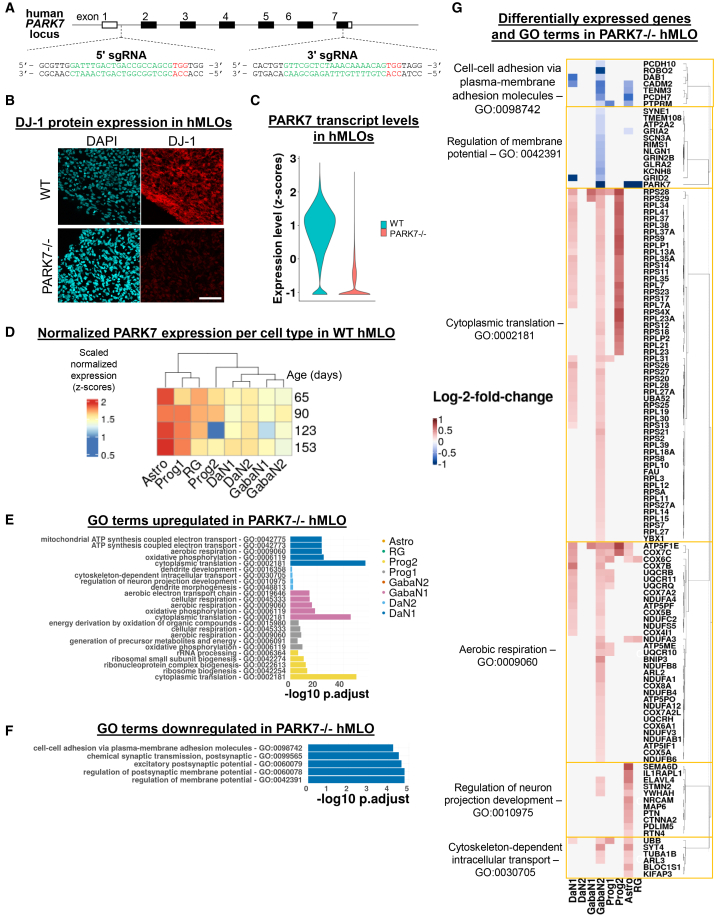
Cell type-dependent molecular perturbations in DJ-1 KO hMLOs (A)Schematic depiction of the CRISPR/Cas9 genome editing strategy for knocking out PARK7, the gene encoding the DJ-1 protein. (B) Immunostaining of day 60 WT and PARK7^−/−^ hMLO for DJ-1 protein. Scale bar: 50μm. (C) Violin plot of normalized expression of PARK7 transcript across all cells (z-scores). (D) Heatmap of scaled log-normalized expression of PARK7 transcript in wildtype (WT) hMLOs. (E) Enrichment scores of the top 5 GO terms upregulated per cell type in PARK7^−/−^ hMLO. (F) Enrichment scores of the top 5 GO terms downregulated in PARK7^−/−^ DaN1. No other downregulated terms were found in the other cell types. (G) Heatmap of log2 fold change expression of genes differentially expressed in PARK7^−/−^ hMLO and their corresponding GO terms. Only GO terms with the largest and least overlapping gene sets are displayed.

Upon confirming that all hMLO cell types express relevant PD genes at varying levels, we next deployed this model to investigate molecular pathophysiology in PD. We created an isogenic knockout of a highly penetrant PD-causing genetic mutation, *PARK7*, which encodes the DJ-1 protein (PARK7^−/−^) (Figures 4A, 4B, and S5A–S5D). We found no difference in midbrain differentiation efficiency of PARK7^−/−^ KO hMLO relative to WT, as determined by expression of key markers at day 30 and 60 (Figures S5E–S5G). PARK7^−/−^ hMLOs were also comparable to WT hMLOs in size, spatial organization, and cell type proportions at day 60 (Figures S5H–S5K).

Next, we examined the cell type-specific and genome-wide effects of PARK7 knockout. We obtained 11,726 single-cell transcriptomes from isogenic PARK7^−/−^ hMLOs (*n* = 12) at days 70, 90, 120, and 150 (Figures S6A, Table S4). All cell types found in WT hMLOs were represented in PARK7^−/−^ hMLOs (Figure S6A). PARK7 transcript was expressed in all cell types of WT but not in PARK7^−/−^ hMLOs (Figures 4C and 4D). We performed GO enrichment analysis on a total of 762 differentially expressed genes across all clusters. Consistent with previously reported functions of DJ-1 protein in cellular oxidative stress responses, mitochondrial quality control, and mRNA binding,^59^^,^^60^^,^^61^^,^^62^ we observed in PARK7^−/−^ hMLOs an upregulation of pathways relating to mitochondrial activities, cytoplasmic translation, and ribonucleoprotein complex biogenesis in DaN1, GabaN1, Prog1, and Prog2 (Figure 4E). These results imply broad molecular perturbations common to many cell types due to the loss of PARK7.

DaN subtypes displayed more specific molecular changes following PARK7 knockout. PARK7^−/−^ DaN2 showed upregulation of terms related to neurite development, while PARK7^−/−^ DaN1 showed downregulation of genes associated with postsynaptic activity (*GLRA2, GRIN2B, RIMS1, TMEM108, SYNE1*) and membrane potential (*PARK7, KCNH8, GRIA2, SCN3A, ATP2A2*) (Figures 4E–4G). We also found *FOXP2*, a marker of resilient DaNs recently discovered in primates,^63^ downregulated in DaN1 of PARK7^−/−^ hMLOs relative to WT (log2FC = −0.67, *p_adj* < 6.5e-05) (Figure S6B).

### PARK7^−/−^ hMLOs recapitulated key cellular phenotypes of PD

**Figure 5:**
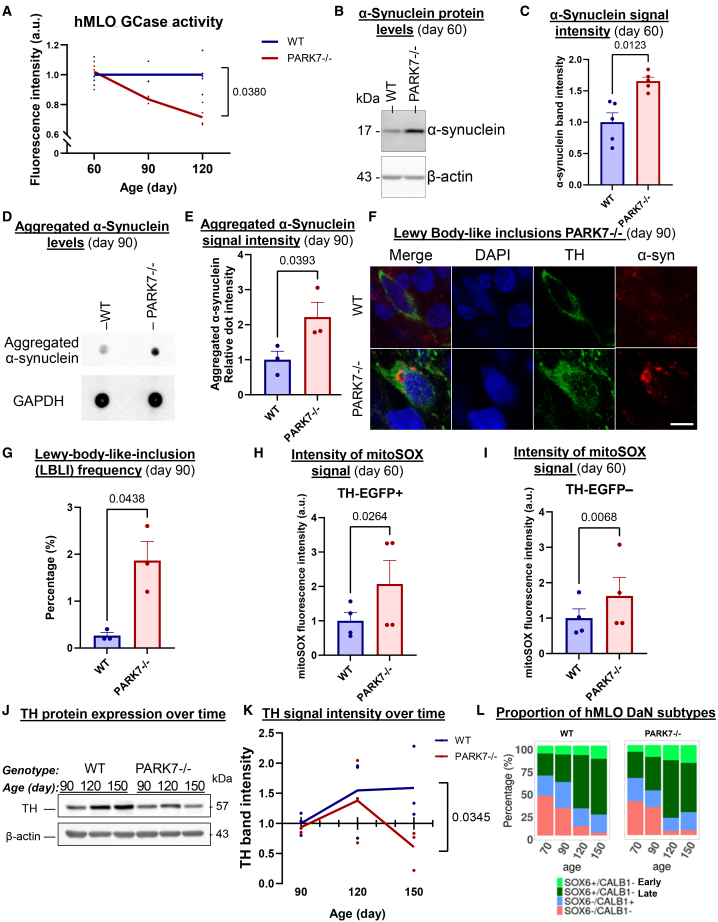
An isogenic PARK7 knockout revealed cell type-dependent molecular perturbations (A) Enzyme activity of GCase in hMLO at indicated timepoints (*p* < 0.05, one-way ANOVA, d60 *n* = 4; d90 *n* = 3; d120 *n* = 5 each for WT and PARK7^−/−^). (B) Western blot for total protein levels of α-synuclein in day 60 hMLOs (*p* < 0.05, Student’s *t* test, *n* = 5). (C) Barplot shows the quantification of (B). Error bars represent SEM. (D) Dot blot analysis of protein lysates from day 90 hMLOs (*p* < 0.05, Student’s *t* test, *n* = 3). (E) Barplot shows the quantification of (D). Error bars represent SEM. (F) Lewy body-like inclusions (LBLIs) displaying halo-like α-synuclein immunoreactivity in TH + neurons identified in PARK7^−/−^ hMLO. Scale bar: 10μm. (G) Barplot shows the quantification of LBLI observed frequency (*n* = 3 independent batches). Error bars represent SEM. (H) Quantification of mean mitoSOX fluorescence intensity in TH + cells on day 60 hMLO (*n* = 3 independent batches, *p* < 0.05, Student’s *t* test). Error bars represent SEM. (I) Quantification of mean mitoSOX fluorescence intensity in TH– cells on day 60 hMLO (*n* = 3 independent batches, *p* < 0.05, Student’s *t* test). Error bars represent SEM. (J) Western blot for TH and β-actin in hMLOs of indicated ages. (K) Quantification of Figure 5J. (*n* = 3 independent batches). (L) Stacked barplot shows SOX6/CALB1 subtype proportions with age in batch-matched WT and PARK7^−/−^ hMLOs.

We next examined the cellular manifestations of PARK7 knockout in hMLOs to determine if the molecular perturbations we found predict cellular phenotypes. Since reduced lysosomal enzyme β-glucocerebrosidase (GCase) activity and α-synuclein accumulation have been linked to PD,^7^^,^^64^^,^^65^^,^^66^ we assayed for these two features. We identified a time-dependent reduction in GCase activity in PARK7^−/−^ hMLOs (Figure 5A) (*p < 0.05*, two-way ANOVA). In contrast, activity levels of a PD-unrelated lysosomal enzyme α-iduronate-2-sulfatase (IDS) were unchanged, confirming a specific GCase deficit (Figure S6C). PARK7^−/−^ hMLOs also exhibited 1.66-fold higher levels of total α-synuclein protein by day 60 (Figures 5B and 5C) (*p < 0.05*, *t* test), consistent with the upregulation of *SNCA* transcript in *PARK7*^−/−^ DaN1 relative to WT (log2FC = 0.62, *p*_adj < 4.3e-05) (Figure S6D). Furthermore, using an antibody established to specifically detect oligomeric and fibrillar α-synuclein forms,^67^^,^^68^ commonly understood to constitute the neurotoxic form of α-synuclein,^68^^,^^69^^,^^70^ we found a 2.22-fold increase in aggregated α-synuclein immunoreactivity in day 90 PARK7^−/−^ hMLOs (Figures 5D and 5E) (*p* < 0.05, *t* test). Consistent with these observations, PARK7^−/−^ hMLOs co-stained for total and aggregated (pS129) forms of α-synuclein confirmed that a fraction of total α-synuclein was phosphorylated at serine 129 (Figure S6E).

We also observed cytoplasmic staining of discrete, packed, and ring-shaped structures immunopositive for α-synuclein, reminiscent of mature Lewy body-like inclusions (LBLI) (Figures 5F and S6F), in approximately 2% of PARK7^−/−^ cells (Figure 5G), consistent with our previous report in the hMLO model of simultaneous GCase loss-of-function and α-synuclein overexpression.^7^

Lastly, using a TH-EGFP reporter line (Figures S6G–S6I), we demonstrated that mitochondrial oxidative stress is increased in both TH+ and TH- cells in PARK7^−/−^ hMLOs relative to WT (*p* < 0.05, *t* test) (Figures 5H and 5I), corroborating the upregulation of GO terms relating to mitochondrial dysregulation in our scRNAseq data (Figure 4E). Collectively, our results suggested concurrent perturbations of multiple cellular processes in PARK7^−/−^ hMLOs, agreeing with current literature on cell-intrinsic molecular functions of DJ-1 protein in healthy and PD conditions.^59^^,^^71^^,^^72^^,^^73^

To find out if PARK7^−/−^ hMLOs display DaN-specific neurodegeneration, we measured protein levels of the DaN marker TH in PARK7^−/−^ hMLOs and identified a decrease in TH protein levels upon long-term culture relative to WT hMLOs (*p* < 0.05, two-way ANOVA) (Figures 5J and 5K). Consistent with this finding, the SOX6+/CALB1- Late subtype comprised 54.9% of all ventral DaNs in PARK7^−/−^ hMLOs compared to 62.0% in batch-matched WT hMLOs, as shown by scRNAseq (Figure 5L). That this reduction in TH levels became apparent only at day 150 despite comparable expression of ventral midbrain progenitor markers and of TH at earlier timepoints (Figure S5F) implicates neurodegeneration as a likely process. The delayed emergence of neurodegeneration relative to molecular deficits such as lysosomal dysfunction and α-synuclein pathology marked by LB formation also agrees with the working hypothesis of temporal progression of classical PD.^74^^,^^75^^,^^76^ Together, our results demonstrate the utility of the hMLO system in modeling and investigating both molecular and cellular pathologies of PD.

## Discussion

Here, we conducted spatiotemporal single-cell analysis of a heterogeneous yet physiologically relevant hMLO model to evaluate its potential in modeling disease progression and selective vulnerability in PD. We showed that our hMLOs not only recapitulated broad cellular diversity and regional patterning of the human midbrain, but also produced distinct DaN subtypes that segregate along a key developmental transcriptomic axis defined by *SOX6* and *CALB1* expression.^12^ This axis of variation remains into adulthood and is an important feature of PD pathophysiology as it demarcates the selective vulnerability and relative resilience of midbrain DaNs in PD.^16^ Although our hMLO DaN subtypes resemble adult midbrain DaN classes, they do not further segregate into the more granular DaN subtypes that are reported to be in adult human midbrain.^16^^,^^39^ This difference is potentially attributable to the short duration of *in vitro* organoid culture relative to adult human development, since human fetal midbrain DaNs also do not display finer subtypes.^12^ Future work can focus on transcriptomic profiling of DaN subtypes in extended hMLO culture, while advancing methods to enhance these features and further improve the physiological relevance of this model system.

The differential enrichment of PD-associated genes in different hMLO cell populations largely agrees with genetic patterns observed in corresponding cell types of postmortem midbrain.^16^ Notably, certain sPD risk loci were relatively enriched in progenitors and less mature DaN subtypes, indicating that sPD genes may participate in cellular development and differentiation.^54^ Lineage tracing of these early populations in hMLOs, combined with perturbative assays, may provide more definitive answers on the impact of these fPD and sPD genes on the cell types that become selectively vulnerable later on. Future investigations comparing iPSC-derived hMLOs from sPD patients with those from healthy controls may also provide additional insights relevant to common forms of PD.

We demonstrated that an isogenic knockout of PARK7 recapitulated key pathological phenotypes of PD. This is the first report of spontaneous LBLI emergence and subsequent neurodegeneration in an *in vitro* fPD model from a single gene mutation, underscoring the system’s maturity in capturing PD hallmarks.^6^ The α-synuclein LBLI phenotype concurs with our previous report in hMLOs harboring dual *GBA1*^−/−^ and *SNCA*-overexpressing mutations,^7^ indicating converging pathogenic pathways across various forms of PD.^77^

Our work, being mostly neuronal-centric, is also complementary to the growing body of evidence of the role of astrocytic DJ-1 in PD.^78^^,^^79^ Notably, the upregulation of mitochondrial activities driven by PARK7 knockout is indiscriminate across many hMLO cell types. Hence, while altered mitochondrial function is prevalent in PD and thought to contribute to the selective loss of DaNs with inherently high bioenergetic demand, it may not be the primary driver of DaN vulnerability. Conversely, the downregulation of GO terms for membrane potential and postsynaptic activity specifically in DaN1 upon PARK7 knockout highlights synaptic pathophysiology as a key contributor instead. Indeed, DJ-1 protein is known to be expressed in neurite terminals and participates in synaptic function^80^ while a progressive loss of neurite extension precedes neurodegeneration in PARK7^−/−^ mouse DaNs.^72^ We anticipate future studies on synaptic pathophysiology in hMLO models will provide more insights into the determinants of selective DaN degeneration in PD.

### Limitations of the study

Our hMLO model faces inherent limitations common to most organoid models,^48^^,^^81^^,^^82^ such as immaturity and the lack of vascularization and circuit integration across brain regions. Despite these limitations, our hMLOs were able to model important aspects of the human midbrain and produce key pathological features of PD, such as LBLIs and neurodegeneration. Recent advances in organoid implantation into the mouse brain,^83^ and assembloid model systems generated via *in vitro* fusion of distinct region-specified organoids,^84^ are potential strategies that could be used to address these physiological limitations and further promote manifestations of PD phenotypes that our hMLOs already exhibit, thereby empowering mechanistic investigations into disease mechanisms.

## Resource availability

### Lead contact

Jinyue Liu (liu_jinyue@a-star.edu.sg).

### Materials availability

The cell lines used in this study are available upon reasonable request from N.H.H. (nghh@a-star.edu.sg).

### Data and code availability

• The sequencing and spatial data generated in the current study have been uploaded to the NCBI GEO repository (GSE271115, GSE271116, GSE271118) (see key resources table).
• Analysis pipelines developed in this work are publicly available at https://doi.org/10.5281/zenodo.18159210.
• Data presented in this study is available upon reasonable request from L.J. (liu_jinyue@a-star.edu.sg).

## Acknowledgments

This research is supported by the 10.13039/100021122Singapore Ministry of Health’s National Medical Research Council under its Open Fund-Large Collaborative Grant (Project ID: MOH-000888-00), and the 10.13039/501100001348Agency for Science, Technology and Research (A∗STAR). The authors would also like to acknowledge all past and current members from the labs of Professor Ng Huck Hui and Dr Liu Jinyue, especially Dr Tran Hoang-Dai and Ms Chang Ya Yin, as well as the Next Generation Sequencing Platform (NGSP) at GIS, for their invaluable contributions to this study.

## Author contributions

Conceptualization: J.J.X., H.H.N., and J.Y.L.; methodology: J.J.X., M.V., T.W.S., K.L.L., H.H.N., and J.Y.L.; investigation: J.J.X., T.Y.S., A.X.S., and L.Y.; data curation: M.V. and J.Y.L.; formal analysis: M.V., J.J.X., and J.Y.L.; software: M.V.; validation: Q.D.; resources: H.H.N. and J.Y.L.; visualization: J.J.X., M.V., Q.D., and J.Y.L.; writing – original draft: J.J.X., M.V., and J.Y.L.; writing – review and editing: M.V., Q.D., A.X.S., T.W.S., K.L.L., E.K.T., H.H.N., and J.Y.L.; supervision: T.W.S., K.L.L., E.K.T., H.H.N., and J.Y.L.; funding acquisition: H.H.N. and J.Y.L.; project administration: H.H.N. and J.Y.L.

## Declaration of interests

The authors declare no competing interests.

## Declaration of generative AI and AI-assisted technologies in the writing process

During the preparation of this work, the authors used ChatGPT (OpenAI, https://openai.com/chatgpt) for grammatical corrections, sentence refinements, and code refactorization advice. After using this tool/service, the authors reviewed and edited the content as needed and take full responsibility for the content of the published article.

## STAR★Methods

### Key resources table

**Table:** 

REAGENT or RESOURCE	SOURCE	IDENTIFIER
**Antibodies**		

DAPI	Sigma-Aldrich	D9542
Thioflavin S staining	Sigma-Aldrich	T1892
Anti-TH, monoclonal	Immunostar	22491
Anti-GABA, polyclonal	Sigma-Aldrich	A2052
Anti-MAP2, polyclonal	abcam	Ab5392
Anti-α-synuclein, monoclonal	BD Bioscience	610787
Anti-α-Synuclein Phospho S129 (MJF-R13), monoclonal	Abcam	ab168381
Anti-Aggregated α-synuclein (5G4)	Millipore	MABN389
Anti-CC3, monoclonal	Cell Signaling	9664S
Anti-Vimetin, monoclonal	BD Bioscience	550513
Anti-FOXA2	abcam	Ab108422
Anti-OTX2	Neuromics	GT15095
Anti-DJ-1	Santa cruz	Sc-55572
Anti-LMX1A, polyclonal	Santa cruz	Sc-54273
Anti-TUJ1, monoclonal	Biolegends	801213
WesternBright Sirius-Femtogram HRP Substrate	Advansta	K-12043-D20
Azure c600 Gel Imaging System	On site	AZI600-01
Leica TCS SP8 confocal microscope	On site	N/A
BD FACSAria II (flow cytometer)	On site	N/A
Lonza 4D-Nucleofector© X and core Unit	Onsite	AAF-1003X, AAF-1003B

**Chemicals, peptides, and recombinant proteins**		

mTeSR1	StemCell Technologies	#85850
DMEM/F12	Invitrogen	11330032
B27 without Vitamin A	Invitrogen	12587010
N2 Supplement	Invitrogen	17502048
GlutaMAX	Invitrogen	35050061
Non-essential amino acid	Invitrogen	11140–050
Beta-mercaptoethanol	Invitrogen	21985023
Rock Inhibitor thiazovivin	Tocris	3845
Heparin	Sigma-Aldrich	9041-08-1
SB431542	Stemgent	04–0010
Noggin	ProSpec	CYT-475
CHIR99021	Reagents Direct	27-H76
SHH-C25II	R&D Systems	464-SH-025/CF
FGF8	R&D Systems	423-F8-025/CF
GDNF	Peprotech	450–10
Matrigel©	Sigma-Aldrich	354277
insulin	Sigma-Aldrich	11061-68-0
laminin	Sigma-Aldrich	L4544
purmophormine	Stemgent	04–0009
BDNF	Peprotech	450–02
ascorbic acid	Sigma-Aldrich	A4544
db-cAMP	Sigma-Aldrich	D0627
MitoSOX Red	Thermo Fisher	M36008
RIPA buffer	Pierce/Thermo Scientific	89900
4MU-glucopyranoside	Sigma-Aldrich	18997-57-4
4MU-sulfate potassium salt	Sigma-Aldrich	M7133

**Critical commercial assays**		

Papain Dissociation system	Worthington, Columbus, OH	LK003150
P1 Primary Cell 4D-Nucleofector® X	Lonza	V4XP-1024
Bradford reagent	BioRad	5000201

**Deposited data**		

hMLO bulk RNA sequencing	This paper	GSE271115
hMLO Single-cell RNA sequencing	This paper	GSE271116
hMLO Multiplexed Error Robust Fluorescence *In Situ* Hybridization	This paper	GSE271118
Single-cell RNA sequencing fetal midbrain	Github of Sten Linnarsson lab	https://github.com/linnarsson-lab/developing-human-brain
Single-cell RNA sequencing fetal midbrain	NCBI Gene expression Omnibus	https://www.ncbi.nlm.nih.gov/geo/query/acc.cgi?acc=GSE76381
Single-cell RNA sequencing ventral midbrain organoid	NCBI Gene expression Omnibus	https://www.ncbi.nlm.nih.gov/geo/query/acc.cgi
Single-nucleus RNA sequencing adult human midbrain	Github of Sten Linnarsson lab	https://github.com/linnarsson-lab/adult-human-brain
Single-nucleus RNA sequencing adult human substantia nigra dopaminergic neurons	Broad Institute Single Cell Portal	https://singlecell.broadinstitute.org/single_cell/study/SCP1768/

**Experimental models: Cell lines**		

H9 human embryonic stem cells	WiCell Research Institute	WA09
H9 PARK7 KO	This paper	N/A
TH-EGFP	This paper	N/A
TH-EGFP DJ-1 KO	This paper	N/A

**Oligonucleotides**		

gRNAs for PARK7 KO (5′-GATTTGACTGACCGCCAGCGTGG-3′ and 5′-GTTCGCTCTAAACAAAACAGTGG-3′)	This paper	N/A
sgRNA (5′-AGTGCCATTGGCTAGGTGCACGG -3′) targeting exon 14 of the TH gene	This paper	N/A

**Recombinant DNA**		

lentiCRISPRv2 plasmid	Addgene	#52961

**Software and algorithms**		

GraphPad Prism 10	GraphPad	N/A
Fiji	ImagJ	https://imagej.net/Fiji/Downloads
RStudio	RStudio	https://www.rstudio.com/products/rstudio/download/
CellRanger 7.0.1	10x Genomics	https://www.10xgenomics.com/support/software/cell-ranger/latest/release-notes/cr-release-notes
Cellpose	Pachitariu and Stringer^85^	github.com/mouseland/cellpose
kb-python	Sullivan et al.^86^	github.com/pachterlab/kb_python
scVelo	Bergen et al.^87^	github.com/scverse/scvelo
Seurat	Stuart et al.^88^	github.com/satijalab/seurat
UCell	Andreatta and Carmona^89^	github.com/carmonalab/UCell
DESeq2	Love et al.^90^	bioconductor.org/packages/DESeq2
Kendall	McLeod 2022	cran.r-project.org/package=Kendall
ggplot2	Wickham 2016	github.com/tidyverse/ggplot2
ggridges	Wilke 2025	github.com/wilkelab/ggridges
pheatmap	Kolde^91^	github.com/raivokolde/pheatmap
MASS	Venables and Ripley^92^	cran.r-project.org/package=MASS
viridis	Garnier et al. 2023	github.com/sjmgarnier/viridis
clusterProfiler	Wu et al.^93^	github.com/YuLab-SMU/clusterProfiler
fdrtools	Strimmer^94^	github.com/cran/fdrtool
igraph	Csárdi et al.^95^	github.com/igraph/rigraph
ComplexHeatmap	Gu et al.^96^	github.com/jokergoo/ComplexHeatmap
Harmony	Korsunsky et al.^97^	github.com/immunogenomics/harmony
tximport	Soneson et al.^98^	github.com/thelovelab/tximport
FlowJo 10.7	FLowJo, LLC	NA
Code Repository for this Publication	This paper	DOI: https://doi.org/10.5281/zenodo.18159210

### Experimental model and study participant details

#### hESC culture and cell line generation

Human embryonic stem cells (hESCs) were maintained as previously reported.^7^ Briefly, we used feeder-free culture in 6-well tissue culture plates in mTeSR1 (Stem Cell Technologies) at 37°C in a humidified CO_2_ incubator, with daily media change. hESCs were maintained for a maximum of 20 passages and tested monthly to confirm absence of mycoplasma contamination. Cell lines were generated via CRISPR/Cas9 genome editing using similar methods as previously reported,^7^ using gRNAs (5′-GATTTGACTGACCGCCAGCGTGG-3′ and 5′-GTTCGCTCTAAACAAAACAGTGG-3′) cloned into the lentiCRISPRv2 plasmid (Addgene plasmid #52961) to mediate knockout of the *PARK7* gene. The TH-EGFP reporter hESC line was generated using the sgRNA (5′-AGTGCCATTGGCTAGGTGCACGG -3′) targeting exon 14 of the TH gene, together with a plasmid donor carrying the homology-directed repair (HDR) template with T2A-EGFP sequence.

#### hMLO generation

hMLOs were generated as previously published.^6^^,^^7^ In brief, TrypLE-Express-dissociated hPSCs were seeded into a V-bottom 96-well culture plate (Sumitomo Bakelite) in neuronal induction media with the ROCK inhibitor thiazovivin, which was removed after 24 h. Neuronal induction medium, used for the first three days, contained DMEM/F12:Neurobasal (1:1), N2 supplement (1:100), B27 without vitamin A (1:50), GlutaMAX (1%), minimum essential media-nonessential amino acid (1%), and β-mercaptoethanol (0.1%) (all Invitrogen) supplemented with 1 μg/mL heparin (Sigma-Aldrich), 10 μM SB431542 (Stemgent), 200 ng/mL Noggin (Prospec), and 0.8 μM CHIR99021 (Reagents Direct). On day 3, 100 ng/mL SHH-C25II and 100 ng/mL FGF8 (both R&D Systems), along with 0.5 μM purmorphamine (Stemgent), were added for midbrain patterning. On day 7, media was completely removed and organoids were embedded in reduced growth factor Matrigel and left to solidify in a 37°C incubator for 30 min, then grown in tissue growth induction medium containing Neurobasal, 1:100 N2 supplement, 1:50 B27 without vitamin A, 1% GlutaMAX, 1% minimum essential media-nonessential amino acid, and 0.1% β-mercaptoethanol supplemented with 2.5 μg/mL insulin (Sigma-Aldrich), 200 ng/mL laminin (Sigma-Aldrich), 100 ng/mL SHH-C25II, and 100 ng/mL FGF8. On day 8, hMLOs were transferred using a wide-bore pipette tip into low-attachment 6-well-plates (Costar) in final differentiation media, consisting of Neurobasal medium, N2 supplement (1:100), B27 without vitamin A (1:50), GlutaMAX (1%), minimum essential media-nonessential amino acid (1%), β-mercaptoethanol (0.1%), 10 ng/ml BDNF (Peprotech), 10 ng/mL GDNF (Peprotech), 100 μM ascorbic acid (Sigma-Aldrich), and 125 μM db-cAMP (Sigma-Aldrich), and were cultured on orbital shakers for maintenance.

### Method details

#### Papain dissociation of hMLOs

Following manufacturer’s protocol, 50 units of papain enzyme (Worthington) was activated with 1 mM L-cysteine and 0.5 μM EDTA in Earle’s Balanced Salt Solution (EBSS) (Thermo Fisher), and DNase I (Worthington) added. hMLOs were finely chopped with a scalpel and then resuspended in papain enzyme mix. The slurry was incubated at 37°C, 5% CO2 for 45 min on a rotator, and then triturated. Cells were spun down at 300xg for 5 min and washed twice in EBSS containing ovalbumin (Worthington) and BSA.

#### Cryosectioning

hMLOs were fixed in 4% paraformaldehyde at 4°C overnight, washed in PBS twice, and then transferred to 30% sucrose overnight at 4°C. hMLOs were embedded in Optimal Cutting Temperature (O.C.T.) compound (Tissue-Tek), then flash-frozen in a slurry of dry ice and ethanol. For immunofluorescence and Thioflavin S staining, 16 μm sections were cut on a Leica cryostat, and 10 μm sections for MERFISH.

#### MERFISH encoding probe design

The MERFISH encoding library used here was designed as previously described.^42^^,^^43^ In brief, a unique binary barcode, drawn from a 16-bit, Hamming-Distance-4 (HD4), Hamming-Weight-4 encoding scheme, was assigned to each of the 136 genes, along with 4 extra ‘blank’ barcodes not assigned to any genes to provide a measure of the false-positive rate. Target regions were designed to (1) have a GC content between 35 and 75%, (2) a melting temperate between 60 and 80°C, (3) a gene specificity index >0.9 (it can range from 0 being non-specific to 1 being most specific) and (4) no homology longer than 15 nt to rRNA or tRNAs. A minimum of 64 qualifying target regions was required to ensure robust detection by MERFISH. MERFISH readout probes were also designed and synthesized as described in the original publication.^42^

#### Tissue preparation for MERFISH

Following cryosectioning, sections were fixed for 1 min with ice-cold 4% PFA then 3:1 MeOH:acetic acid for 5 min, with 3 × 5 min washes with cold PBS in between. Air-dried coverslips were stored at −20°C. Coverslips were thawed and permeabilized with 70% EtOH at 4°C for 48 h, and incubated with wash buffer for 2 h in a 47°C incubator, before 48 h incubation at 47°C with 75–100 μM of encoding probe library. Before imaging, coverslips were washed twice with wash buffer for 15 min at 47°C, stained with 4′,6-diamidino-2-phenylindole (DAPI), then washed twice with 2X saline sodium citrate buffer. Imaging was performed on a commercial platform using a custom-built microscope.

#### Protein extraction, and western blotting

hMLOs were lysed in ice-cold RIPA lysis buffer (Pierce Thermo Scientific), with protease inhibitor, phosphatase inhibitor and PMSF (1 mM) added fresh. 100 μL lysis buffer was added to each tube containing two to three organoids. Lysis was aided by trituration using a pipette tip, followed by incubation at 4°C for 20 min. Lysates were then centrifuged at 10,000xg, 4°C for 20 min. Supernatant containing the protein extract was collected into a new tube and stored at −80°C for further use. Protein concentration was measured using Bradford reagent (BioRad). 30–50 μg of protein lysates were mixed with 1X sample buffer and denatured at 98°C for 8 min, then loaded onto 10% SDS-PAGE gels. Gels were run at 100V, and transferred to nitrocellulose membranes by wet transfer at 200 mA for 90 min. For α-synuclein probing, membranes were fixed with 0.4% paraformaldehyde for 30 min before blocking. Membranes were blocked using 5% milk in TBS +0.1% Tween 20 for 1 h at room temperature with rotation. Primary antibodies were incubated overnight at 4°C with rotation; the following day, membranes were washed with TBST thrice, and incubated with secondary antibodies for 1 h at room temperature. Secondary antibodies conjugated to horseradish peroxidase were developed using WesternBright Sirius-Femtogram HRP Substrate (Advansta, K-12043-D20); secondary antibodies conjugated to NIR were imaged on the Azure c600 Gel Imaging System. Quantification was performed using ImageJ software.

#### Lysosomal enzyme activity assay

Artificial enzyme substrates were used to determine lysosomal enzyme activity as previously described^7^: 4MU-glucopyranoside and 4MU-sulfate potassium salt (Sigma-Aldrich) for GCase and a-i-2-sulfate respectively. hMLOs were harvested in activity assay buffer (0.25% Triton X-100, 0.25% [wt/vol] taurocholic acid, and 1 mM ethylenediaminetetraacetic acid in citrate/phosphate buffer [pH 5.4]), and 10 μg protein was added to 10 μL of 10% BSA followed by 20 μL of 5 mM enzyme-substrate. After 30 min incubation at RT, the reaction was stopped by adding an equal amount of 1M glycine, pH 12.5. Samples were loaded onto 96-well plates, and the fluorescence signal was quantified at Ex/Em 365/445 (top read).

#### Immunofluorescence, staining and imaging

Slides were dried on a heat block and washed with PBS to remove OCT. 100 μL of permeabilization/blocking buffer (1.5% BSA +0.5% Triton X-100 in PBS) was added to slides and incubated for 1 h at room temperature. Primary antibody diluted in blocking buffer was then added and slides were placed at 4°C overnight. The next day, primary antibody was removed and slides were washed in PBST for 3 × 5 min. Fluorophore-conjugated secondary antibody was added to the slides and incubated at room temperature for 1 h in the dark, then washed in PBS for 3 × 5 min. Slide was counterstained with DAPI for 7 min, washed, dried, and mounted with FluorSave Reagent (Sigma-Aldrich). Thioflavin S staining was performed by adding 0.025% Thioflavin S in 50% ethanol to the hMLO sections, then incubated for 15 min at RT. Images were taken using a Leica TCS SP8 confocal microscope.

#### Measuring mitochondrial oxidative stress

1-2 million dissociated cells from hMLOs were incubated with MitoSOX Red (5 μM, ThermoFisher) for 30 min at 37°C, 5% CO2. Cells were washed twice in PBS and resuspended in 2% FBS in PBS with DAPI. Flow cytometry was performed on the BD FACSAria II (BD Biosciences), and data was analyzed using FlowJo 10.7 (FlowJo, LLC).

#### Immunofluorescence image analysis

Fluorescent image or mitoSOX-positive cells were manually counted and normalised against total nuclei DAPI count. Graphs were made using Graphpad Prism 10 (GraphPad software). Statistical comparison of the means was performed using unpaired Student’s *t* test or one-way ANOVA when appropriate. Significant differences were considered at *p* < 0.05. All statistical analyses were performed on GraphPad Prism 10.

### Quantification and statistical analysis

#### Quality control of hMLO scRNAseq data

Library preparation for single-cell RNA-seq was performed using Chromium Single Cell 3ʹ GEM kit. Digital count matrices were generated from 10X Genomics output and aligned to the GRch38 genome as reference, with intron mode on CellRanger 7.0.1. We removed low sequencing depth cells with less than 1000 genes per cell, but not more than 10000 as well as damaged cells using a cutoff of 10% cell genome mapping to the mitochondrial genome as well as a cutoff of less than 1% mitochondrial gene expression to remove single nuclei. The gene-cell matrix was further filtered by iteratively clustering and removing clusters associated with cell types that are non-physiological for hMLOs. Cortically misdifferentiated cells were removed based on expression of cortical markers *FOXG1*^99^^,^^100^ and *PAX6*^101^^,^^102^ and vascular leptomeningeal cells based on expression of *COL1A1* and *COL1A3* genes.^103^ Choroid plexus cells were removed based on expression of *TTR, TRPM3* and *AQP1*.^104^^,^^105^^,^^106^ Lastly, through iterative clustering, we also removed uninterpretable clusters with lower number of genes per cell in relation to other clusters, and no clear cell-type identity markers expected of cells in the midbrain (defined as expression of *FOXA2, LMX1A*, and clear expression of neurotransmission markers – *GAD2*, or *TH*).

#### Cell-type clustering of hMLO data

After quality control, each sample was normalized using regularized negative binomial regression implemented by Seurat’s SCTransform function (“v2” version).^107^^,^^108^ Mean-variance stabilizing transformation was used to calculate 1075 highly variable genes per sample and then the intersection of 301 highly variable genes in all samples was chosen.^88^ 12 principal components (PCs) were chosen based on the “elbow plot” method. We then combined all objects and performed Principal Component Analysis (PCA) on the 301 variable features. Uniform Manifold Approximation and Projection (UMAP) embedding was calculated on the 12-component embedding, followed by Jaccard index between every cell and its 30-nearest neighbors to construct a shared nearest neighbor (SNN) graph. Louvain clustering was used to partition the graph using resolution = 0.5. 9 cell-type identities were assigned based on differentially expressed genes from the FindAllMarkers function: Progenitors 1 (Prog1), Progenitors 2 (Prog2), Astrocytes (Astro), DaN 1 (DaN1), DaN 2 (DaN2), GABAergic Neurons 1 (GabaN1), GABAergic Neurons 2 (GabaN2), Radial-glia cells (RG). Astro cells were identified after subclustering to isolate cells expressing canonical astrocyte markers *GFAP* and *GJA1*. For visualizing proportion changes over time (Figure S1B), the dataset was downsampled to equal the number of cells between the sequencing batches (5431 cells per batch).

#### Quality control of other scRNAseq datasets

To perform quality control for the scRNAseq fetal midbrain dataset,^12^ metadata and a filtered.h5 object were retrieved from GitHub (https://github.com/linnarsson-lab/developing-human-brain). Only samples acquired using the 10X Genomics v3 chemistry were included as Chromium chemistry was a significant confounding technical factor. Non-physiological cell types for the organoid system (erythrocytes, fibroblasts, microglia, and placode cell classes) were removed. Quality control filtering from the original paper was used,^12^ excluding cells labeled as doublets in the filtered.h5 file and cell clusters marked as doublets and “bad cells” in the cluster-level metadata file. Cells passing quality control were identified using the “ValidCells” metadata column, and gene-level quality control used the “ValidGenes” column.

Another scRNAseq fetal midbrain dataset^11^ was retrieved from NCBI (https://www.ncbi.nlm.nih.gov/geo/query/acc.cgi?acc=GSM2268853). Author-provided cell-level quality control was used. Non-physiological cell types for organoids were removed (labels: “hRN”, “hEndo”, “hMGL”, “Unk”, “hPeric”, “hOPC”, “hOMTN”). Each sample underwent normalization using SCTransform. 999 highly variable genes were identified. After merging the samples, the dataset underwent PCA, SNN graph construction, Louvain clustering, and UMAP latent space creation. 11 principal components (PCs) to project the data into a latent uniform manifold approximation and projection (UMAP) space. Granular labels provided by the authors were manually merged based on proximity in latent UMAP space.

For quality control of scRNAseq data^9^ from ventral midbrain (VM) organoids, we excluded cells expressing fewer than 600 or more than 12,000 genes, those with over 20% mitochondrial genome mapping, and cells with over 30,000 reads per cell. Analysis focused on “standard organoids” aged 60, 90, and 120 days. SCTransform normalization was used, identifying 4,000 highly variable features. PCA, SNN, Jaccard index, Louvain clustering and UMAP latent space were calculated. Cell types were annotated using gene markers identified through the Wilcoxon differential expression test (log2fc.threshold = 0.25; min.pct = 0.25) and gene markers from the publication.^9^ Vascular leptomeningeal cells (VLMCs) were excluded.

The single-nucleus RNA sequencing (snRNAseq) data from the adult human midbrain,^39^ metadata and a filtered.h5 object were obtained from GitHub (https://github.com/linnarsson-lab/adult-human-brain). Cell-level quality control included retaining cells from clusters with “midbrain” among the top three regions or more than 1% of cells assigned to “midbrain”. Cells were required to have “midbrain” in the ROIGroupFine column. Clusters with fewer than 20 cells and doublets (DoubletFinder score <0.3) were excluded. Cells with fewer than 800 UMIs per cell and fewer than 40% unspliced molecules per cell were removed. Cell types labeled “Microglia”, “Ependymal”, “Lower Rhombic Lip”, “Upper Rhombic Lip”, “Vascular”, and “Miscellaneous” were excluded, as well as cell classes indicative of other anatomical regions (“Hippocampal Dentate Gyrus,” “Thalamic Excitatory,” “Hippocampal CA4,” and “Cerebellar Inhibitory”).

Lastly, a snRNAseq dataset^16^ of adult postmortem substantia nigra dopaminergic neurons was retrieved from Broad Institute Single Cell Portal (https://singlecell.broadinstitute.org/single_cell/study/SCP1768/). No additional filtering was done on this dataset. Only neurotypical samples that were FACS for NR4A2 were selected for analysis.

#### TH+ co-transmission analysis

To quantify the proportions of dopaminergic neurons that putatively co-transmit other neurotransmitters (serotonin, GABA and glutamate) in hMLOs and human midbrain, scRNAseq data from three distinct datasets were analyzed. For the hMLO dataset, only wild-type neuronal cells annotated as “DaN1”, “DaN2”, “GabaN1”, and “GabaN2” were included and subsequently downsampled to 4061 cells per sequencing batch to account for batch variability. Sequencing-depth corrected counts obtained via SCTransform (SCT) were utilized, focusing specifically on cells expressing at least one read of the neurotransmitter markers *TH, TPH1,* or *GAD2*, thereby excluding cells negative for all three markers. We also excluded TH + cells that were co-transmitting more than one neurotransmitter (e.g., TH+/TPH1+/GAD2+). The overlap between cells positive for each neurotransmitter marker was then assessed by counting shared barcode identifiers and visualized as proportions in a stacked barplot. A similar approach was applied to the adult midbrain dataset,^39^ restricting analysis to neuronal cell types annotated as “Splatter” and “Midbrain-derived inhibitory neurons”, with each sample downsampled to match the smallest donor group of 22,530 cells, thus mitigating previously reported donor-specific batch effects. The fetal midbrain dataset^12^ included V3 Chromium chemistry neuronal cells labeled “Neuron”, “Neuroblast”, and “Neuronal IPC”, and was analyzed similarly without downsampling due to the absence of significant batch effects.

#### Correlational analysis between cell classes

We calculated 2000 highly variable features shared by all samples from each dataset using mean-variance stabilizing transformation. Next, we calculated SCT-normalized Pearson residuals of each cell class using these 2000 highly variable features.^88^ Lastly, we calculated pairwise Spearman’s rho statistic using base R function cor() to estimate rank-based measure of association between cell classes.

#### Subregional fetal midbrain marker discovery

To assign fetal regional identity to hMLO cells, we selected seven samples with dissection information from a fetal midbrain dataset (labeled “ventral midbrain” or “dorsal midbrain”) across both V2 and V3 Chromium chemistries.^12^ We then performed pseudo-bulk differential expression analysis using the DESeq2 package,^90^ regressing out chemistry effects in the design formula (design = ∼Chemistry + Subregion) for the Wald test. Genes that did not reach statistical significance (p.adj <0.05) or had low overall expression (BaseMean <200) were excluded, and fetal ventral and dorsal markers were defined as those with an absolute log_2_-fold-change greater than 3.

Next, we computed module scores for ventral and dorsal gene sets using UCell.^89^ UCell ranks genes by their expression in each cell and calculates the Mann–Whitney U statistic for the gene module of interest. Counts normalized for sequencing depth via SCTransform (SCT counts) were used as input. After module scoring, we derived a ventrality index by taking the ratio of ventral to dorsal module scores. Cells with ventrality index above 1 were classified as ventral, those below 1 as dorsal, and cells with both module scores below the 25th quantile were designated as ambiguous.

#### Ventral/dorsal proportion changes over time

Samples from closely adjacent timepoints were merged for ease of interpretation (days 65 and 70 as “day 65”, days 99 and 105 as “day 102”, days 120 and 126 as “day 123”, and days 150 and 156 as “day 153”). The Mann-Kendall test was applied to assess the statistical significance of changes in ventral/dorsal cell type proportions over time (Figure 1G). Analysis was implemented using *Kendall* package in R.

#### MERFISH preprocessing and spot assignment

Images of individual hybridization rounds were merged by z stack and stitched to get joint X-Y coordinates per organoid section. RNA spots were decoded using a custom script using HD4-logic outlined above. Nuclei segmentation of DAPI staining was performed using CellPose v2.0.^85^ Nuclei were then dilated by 10 pixels or 14 pixels to create cytoplasmic signal, depending on the resolution used in the imaging round. Spots were then assigned to cells by checking if a spot exists within the cell mask. Spots assigned to unsegmented areas were filtered out.

#### Validating MERFISH spot decoding

Bulk RNA-sequencing was used to validate accuracy of the MERFISH spot decoding [see below “Quality control in MERFISH”]. Library preparation for bulk RNA-seq was performed by Novogene AIT Singapore Bulk RNA-seq datasets were analyzed using the nf-core/RNA-Seq pipeline version 1.4.2. In brief, reads were quality-checked using FASTQC to ensure sequencing quality, then processed for adaptor and low read quality trimming (TrimGalore v0.6.4). Transcripts were quantified using tximport package^98^ that quantifies transcript abundance of different reads using STAR transcript-quantification output (v2.6.1, GRch38 used as a reference).

#### Quality control in MERFISH

We removed cells whose nucleus and cell area were in the 2.5% and 97.5% quantile for either of these metrics. Secondly, we removed “blank” genes. Thirdly, we filtered cells expressing fewer than 10 genes per cell and less than 13 reads per cell. Correspondence to bulk RNAseq was evaluated by calculating the linear correlation coefficient between MERFISH mRNA transcript count with bulk RNAseq FPKM for each gene in the MERFISH panel.

#### MERFISH cell-type annotation

The MERFISH dataset was clustered using an iterative re-normalization and graph-based clustering approach. Different MERFISH fields of view were merged per organoid and each individual organoid data was then normalized using the SCTransform (v2).

In the initial clustering round, 87 overlapping genes identified between scRNAseq cluster markers and the MERFISH spatial gene library guided PCA dimensionality reduction. This clustering employed 6 principal components (PCs) in dimensionality reduction, a resolution of 0.8 in Louvain clustering, and Wilcox-based differential expression analysis using FindAllMarkers() (min.pct = 0.25, logfc.threshold = 0.25, assay = “SCT”, slot = “data”). The analysis yielded 40 unique cluster gene markers. A non-dopaminergic neuronal cluster defined by markers *DCX, STMN2, NSG1, STMN4*, and absence of *LMX1A* and *ENO2* was excluded, as it likely represented MERFISH-specific gene dropout artifacts rather than a genuine biological population.

In the second clustering round, each organoid was individually re-normalized (136 genes, 120,448 cells) with SCTransform (v2), followed by PCA and clustering using 40 highly variable genes, 8 PCs, and a resolution of 0.8. Final 7 cell-type identities were assigned based on cluster-specific DEGs identified through Wilcox testing, employing the same thresholds as the initial round. The cell-type or interior/exterior proportions were averaged over images coming from the same organoids. The organoids were downsampled to the smallest organoid – 41604 in the case of WT organoids and 8957 in the case of DJ-1 KO. Two-sided Wilcoxon ranked-sum tests were used for proportion analyses.

#### Creating interior/exterior division in hMLO

We first searched for which genes from our spatial library are related to glycolysis and hypoxia and identified 6 genes: *P4HA1, STC2, PGK1, VEGFA, EGLN3, PFKP*. We then calculated an average expression score of these 6 genes for each cell to create a metabolic index. We binarized the cells as either “interior” or “exterior” at the 60^th^ percentile of the metabolic index histogram. For modeling cell-type proportion differences between exterior/interior (Figure S3E), we first calculated cell-type proportions in each sample’s interior/exterior, then used a two-sided Wilcoxon ranked-sum test to estimate the significance between interior/exterior.

#### Validating interior/exterior division in hMLO

We determined the convex hull based on cell centroids using the *chull* function, which draws the smallest possible polygon around the points and calculates the minimal perpendicular distance from each centroid to its boundary. We manually defined high-confidence regions where the mathematically derived convex hull corresponded to the actual organoid edge rather than an internal boundary. Additionally, we eliminated centroids located more than 400 μm from the edge, as these likely represented cells with truncated “real edges” due to limitations in field-of-view sampling. Within these high-confidence areas, we fit a linear loess model using geom_smooth from the *ggplot2* package (span = 1.5) to model the relationship between metagene expression, perpendicular distance to the edge, and cell-type identity, and we visualized the relationship between cell type and distance to the organoid edge with a ridge plot generated by the *ggridges* package.

#### Point density analysis

Following normalization (as described in section “Quality control in MERFISH”), we transformed relabeled DaN1 and DaN2 as DaNs for combined analysis. We then transformed DaN GabaNs spatial distribution to a two-dimensional kernel density estimation using kde2d from MASS package.^92^ The values in this density estimation are then transformed to z-scores for direct comparability and density of GabaNs is subtracted from density of DaNs. The resulting subtracted density matrix is visualized using filled.contour^109^ with DaNs being yellow and GabaNs cell density being blue on the viridis scale.

#### Spatial co-localization analysis

Spatial co-localization analysis was performed for cell centroids of DaNs versus GabaNs. Each tissue map was divided to square grids of 50 μm. We then calculated the Pearson Correlation between gridded cell abundance of DaNs1 and GabaNs. To obtain a null distribution of cellular abundance of DaNs and GabaNs, we randomly shuffled the labels of DaNs and GabaNs for each tissue map 10,000 times. A one-sided test was then applied to derive the *p* value for the observed value relative to the null distribution. Lastly, we performed a paired comparison between the observed Pearson Correlation value of DaNs and GabaNs and the mean of the 10,000 permutated Pearson Correlation values that we define as NULL. Paired Wilcoxon ranked sum test was used to indicate statistical significance between in this comparison.

#### Familial PD (sPD) gene visualization

17 genes were selected from a comprehensive study of different rare familial PD (fPD) cases.^50^ Gene expression heatmap of fPD genes was displayed by taking sequencing-depth corrected counts (SCT.counts) which were then averaged per cell type and log1p normalized. The resulting values were then transformed to z-scores per column and then both rows and columns were hierarchically clustered based on Euclidian distance.^103^ Non-zero expression was determined by inspecting the number of counts that were sequencing-depth corrected (SCT, counts).

#### Sporadic PD (sPD) risk enrichment

We curated 93 sPD risk genes from 2 meta-genome wide association study (GWAS) for sporadic PD (sPD) in the European^51^ and Asian^52^ populations. We included both genes that were nearest to GWAS loci implicated in PD risk and genes from rare coding variant burden analysis (e.g., *GBA1, LAMB2*). We then defined genes that are above baseline expression level per gene across cell-types by taking all genes above 1 count across all cells for that cell type using sequencing depth corrected counts (SCT, counts). Finally, we performed Fisher’s exact *t* test (alternative = “greater”) between these sPD-associated risk genes and genes expressed above baseline level from each cell type. Bonferroni correction was used to define the alpha value of significance to account for multiple testing. A gene enrichment scores was calculated by taking z-scores across all cells for each gene (RNA, scale.data), scaling across row using pheatmap package and clustering using euclidian distance.^91^ Note that 6 genes were completely not expressed in our dataset (*GBAP1, LINC00693, FAM47E-STBD1, LOC100131289, RABGEF1P1, LOC442028*) and the remaining 87 sPD risk genes are visualized in the heatmap presented in Figure 3B.

#### Discovering ventral DaN subtypes

To discover ventral DaN neuron subtypes, we first selected cells from clusters “Midbrain DaN 1” and “Midbrain DaN 2” which broadly expressed tyrosine hydroxylase (TH)—a rate-limiting enzyme in dopamine biosynthesis—and that were scored as corresponding to a “fetal ventral midbrain” identity (see above). We then renormalized each sample using SCTransform (v2) normalization, and 1120 variable features were calculated per sample. 300 genes highly variable in all samples were selected. This number was selected because it was the smallest number of highly variable features that contained *CALB1* and *SOX6* amongst its variable features which are key markers for dopaminergic neuron vulnerability-resilience to PD.^16^^,^^63^ We then used the regular Seurat pipeline of PCA, UMAP (12 PCs), Jaccard index (12 PCs, k = 30), and SNN graph construction (resolution 0.2). The 4 resulting clusters were labeled using differentially expressed genes from the FindAllMarkers function (min.pct = 0.25, logfc_threshold = 0.5). For UMAP visualizations of WT ventral DaN subtypes (Figures 3D, S4A, and S4C), the object was downsampled to the same number of cells per sequencing batch (1637 cells). For UMAP visualizations of WT ventral DaN subtypes across different timepoints (Figure S4B), the object was downsampled to equal number of cells in all timepoints (390 cells).

#### RNA velocity of ventral DaN subtypes

To investigate the transcriptional dynamics within MLO ventral DaN subtypes, we utilized the scVelo^87^ package for dynamic RNA velocity analysis. (Initially, spliced and unspliced matrices were pseudo-quantified from.fastq.gz files using the kb-python^86^ pipeline with the NCBI_GRCh38 genome reference, consistent with our scRNAseq dataset. Samples were processed using v3 Chromium chemistry whitelist barcodes, except for MLO0, which had 26-base pair reads instead of the standard 28; this difference did not influence subsequent analyses. We then subset the matrices to include only the ventral DaN cells as defined by the scRNAseq analysis. Filtering and normalization of spliced gene counts in the adata file were carried out using the same parameters as the scRNAseq pipeline (min_shared_counts = 30, highly variable genes = 300). Following PCA on 12 principal components, we constructed a nearest neighbor graph and computed first- and second-order moments using 30 nearest neighbors. RNA velocities were finally estimated in “dynamical” mode and visualized with velocity_embedding_stream (arrow_size = 2.5), with vector fields filtered to include only those with a minimum mass of 4. Latent time was calculated on high-confidence genes from RNA velocity calculations using within latent_time (min_likelihood = 0.4). A corresponding smoothed gene expression matrix was calculated using scVelo’s heatmap function by averaging across 30 nearest neighboring cells, arranging the cells by their internal latent time and using n_convolve = 200 parameter for visualization.

#### Label transfer of adult SNpc DaNs identity

We first found out variable features that separate SOX6-CALB1 subtypes within adult SN dataset^16^ alone by running FindAllMarkers (min.pct = 0.2, log2fc.threshold = 2) and overlapping those features with genes expressed in MLO scRNAseq dataset which yielded 363 genes. We then combined the 10 molecular adult SN DaN subtypes to either *SOX6* or *CALB1* subtypes. Next, after merging the adult SNpc and MLO scRNAseq datasets, we constructed a shared latent PCA space using these 363 features and 10 principal components, as determined by the elbow plot method. Harmony-based integration was subsequently applied to correct this shared space, converging after 4 iterations (θ = 5, λ = 1; see Figure S4G).

The label transfer was performed based on the “majority-neighbor” principle. In short, the merged dataset was downsampled to 1,487 cells per unique DaN subtype label across both MLO and Kamath datasets, and we identified 100-nearest neighbors using 10 principal components. Label transfer to MLO cells was performed by assigning, for each cell, the most common Kamath label among its 100-nearest neighbors; cells with tied top neighbors were excluded. Finally, an alluvial plot created with ggplot2 was used to visualize the corresponding cell proportions between MLO and adult SNpc subtypes.

#### Differential expression analysis (cell-types)

We used the pseudo-bulk approach for differential expression using DESeq2.^90^ Using batch-matched samples, Wald *t* test was performed (design = ∼age + genotype + age:genotype). Z-scores from the Wald test were then imputed to fdrtools^94^ to correct overestimated variance of null distribution of the z scores. Genes were then filtered based on statistical significance (<0.05) and base expression level (BaseMean <400) for input to GO analysis. GO analysis was performed using clusterprofiler^93^ using adjusted *p* cutoff 0.05 and *q* cutoff 0.2 (BH correction). We then removed GO terms enriched in fewer than 6 genes.

Since GO terms often share a lot of overlapping genes that go into their gene sets, we further curated the GO terms for which we displayed their respective gene lists. In short, we computed pairwise similarities between each GO term gene set using Jaccard index, used a Jaccard threshold of 0.2 to define “unique GO terms”, clustered GO terms into connected components using igraph package,^95^ and selected the representative GO term with the maximum gene count from each cluster. This gave 6 GO terms and their genes shown in Figure 4G. Heatmaps of log-2-fold-change values for genes from these 6 GO terms were displayed using ComplexHeatmaps.^96^ Columns and rows within each GO term were clustered using Euclidean distance as implemented by ComplexHeatmaps. Individual transcripts of *FOXP2*, and *SNCA* (Figures S6B and S6D) were visualized using log-normalized, scaled and centered data (RNA, scaledata) in DaN1 cells that were downsampled to the same number of cells between genotypes (total 1238 cells).

#### Differential expression analysis (subtypes)

To define the transcriptomic axis of dopaminergic neuron resilience/vulnerability in our MLO system, we wanted to perform a bulk comparison between the two SOX6+ subtypes and the CALB1+ subtype. Specifically, SOX6+/CALB1- Early and SOX6+/CALB1- Late MLO DaN subtypes were combined into a single “*SOX6+”* group (reflecting Kamath’s SOX6 subtype), while the SOX6-/CALB1+subtype was relabeled “*CALB1+*” for clarity; the SOX6-/CALB1- subtype was excluded due to its lack of canonical markers. Using these labels as the basis for our analysis, we performed a pseudo-bulk differential expression analysis on all wild-type samples with DESeq2 using a Wald *t* test (design = ∼age + sequencing_batch + genotype). Genes were filtered based on statistical significance (*p* < 0.05) and base expression (BaseMean <500) before conducting GO analysis with clusterProfiler (adjusted p cutoff = 0.05, q cutoff = 0.2 with BH correction). Finally, the top five GO terms for the *SOX6* and *CALB1* subtypes were visualized using the ggplot2 barplot function.

## References

[1] Kalia L.V., Lang A.E. (2015). Parkinson’s disease. Lancet.

[2] Reumann D., Krauditsch C., Novatchkova M., Sozzi E., Wong S.N., Zabolocki M., Priouret M., Doleschall B., Ritzau-Reid K.I., Piber M. (2023). In vitro modeling of the human dopaminergic system using spatially arranged ventral midbrain–striatum–cortex assembloids. Nat. Methods.

[3] Do Q.B., Noor H., Marquez-Gomez R., Cramb K.M.L., Ng B., Abbey A., Ibarra-Aizpurua N., Caiazza M.C., Sharifi P., Lang C. (2024). Early deficits in an in vitro striatal microcircuit model carrying the Parkinson’s GBA-N370S mutation. npj Parkinson's Dis..

[4] Ryan S.K., Zelic M., Han Y., Teeple E., Chen L., Sadeghi M., Shankara S., Guo L., Li C., Pontarelli F. (2023). Microglia ferroptosis is regulated by SEC24B and contributes to neurodegeneration. Nat. Neurosci..

[5] Barmpa K., Saraiva C., Lopez-Pigozzi D., Gomez-Giro G., Gabassi E., Spitz S., Brandauer K., Rodriguez Gatica J.E., Antony P., Robertson G. (2024). Modeling early phenotypes of Parkinson’s disease by age-induced midbrain-striatum assembloids. Commun. Biol..

[6] Jo J., Xiao Y., Sun A.X., Cukuroglu E., Tran H.-D., Göke J., Tan Z.Y., Saw T.Y., Tan C.-P., Lokman H. (2016). Midbrain-like Organoids from Human Pluripotent Stem Cells Contain Functional Dopaminergic and Neuromelanin-Producing Neurons. Cell Stem Cell.

[7] Jo J., Yang L., Tran H.D., Yu W., Sun A.X., Chang Y.Y., Jung B.C., Lee S.J., Saw T.Y., Xiao B. (2021). Lewy Body–like Inclusions in Human Midbrain Organoids Carrying Glucocerebrosidase and α-Synuclein Mutations. Ann. Neurol..

[8] Kim H., Park H.J., Choi H., Chang Y., Park H., Shin J., Kim J., Lengner C.J., Lee Y.K., Kim J. (2019). Modeling G2019S-LRRK2 Sporadic Parkinson’s Disease in 3D Midbrain Organoids. Stem Cell Rep..

[9] Fiorenzano A., Sozzi E., Birtele M., Kajtez J., Giacomoni J., Nilsson F., Bruzelius A., Sharma Y., Zhang Y., Mattsson B. (2021). Single-cell transcriptomics captures features of human midbrain development and dopamine neuron diversity in brain organoids. Nat. Commun..

[10] Zagare A., Barmpa K., Smajic S., Smits L.M., Grzyb K., Grünewald A., Skupin A., Nickels S.L., Schwamborn J.C. (2022). Midbrain organoids mimic early embryonic neurodevelopment and recapitulate LRRK2-p.Gly2019Ser-associated gene expression. Am. J. Hum. Genet..

[11] La Manno G., Gyllborg D., Codeluppi S., Nishimura K., Salto C., Zeisel A., Borm L.E., Stott S.R.W., Toledo E.M., Villaescusa J.C. (2016). Molecular Diversity of Midbrain Development in Mouse, Human, and Stem Cells. Cell.

[12] Braun E., Danan-Gotthold M., Borm L.E., Lee K.W., Vinsland E., Lönnerberg P., Hu L., Li X., He X., Andrusivová Ž. (2023). Comprehensive cell atlas of the first-trimester developing human brain. Science.

[13] Klar A., Baldassare M., Jessell T.M. (1992). F-spondin: a gene expressed at high levels in the floor plate encodes a secreted protein that promotes neural cell adhesion and neurite extension. Cell.

[14] Kittappa R., Chang W.W., Awatramani R.B., McKay R.D.G. (2007). The foxa2 gene controls the birth and spontaneous degeneration of dopamine neurons in old age. PLoS Biol..

[15] Kriks S., Shim J.W., Piao J., Ganat Y.M., Wakeman D.R., Xie Z., Carrillo-Reid L., Auyeung G., Antonacci C., Buch A. (2011). Dopamine neurons derived from human ES cells efficiently engraft in animal models of Parkinson’s disease. Nature.

[16] Kamath T., Abdulraouf A., Burris S.J., Langlieb J., Gazestani V., Nadaf N.M., Balderrama K., Vanderburg C., Macosko E.Z. (2022). Single-cell genomic profiling of human dopamine neurons identifies a population that selectively degenerates in Parkinson’s disease. Nat. Neurosci..

[17] Panman L., Papathanou M., Laguna A., Oosterveen T., Volakakis N., Acampora D., Kurtsdotter I., Yoshitake T., Kehr J., Joodmardi E. (2014). Sox6 and Otx2 Control the Specification of Substantia Nigra and Ventral Tegmental Area Dopamine Neurons. Cell Rep..

[18] Andersson E., Tryggvason U., Deng Q., Friling S., Alekseenko Z., Robert B., Perlmann T., Ericson J. (2006). Identification of intrinsic determinants of midbrain dopamine neurons. Cell.

[19] Duarte S., Viedma-Poyatos Á., Navarro-Carrasco E., Martínez A.E., Pajares M.A., Pérez-Sala D. (2019). Vimentin filaments interact with the actin cortex in mitosis allowing normal cell division. Nat. Commun..

[20] Bonilla S., Hall A.C., Pinto L., Attardo A., Götz M., Huttner W.B., Arenas E. (2008). Identification of midbrain floor plate radial glia-like cells as dopaminergic progenitors. Glia.

[21] Miranda-Negrón Y., García-Arrarás J.E. (2022). Radial glia and radial glia-like cells: Their role in neurogenesis and regeneration. Front. Neurosci..

[22] Li L., Medina-Menéndez C., García-Corzo L., Córdoba-Beldad C.M., Quiroga A.C., Calleja Barca E., Zinchuk V., Muñoz-López S., Rodríguez-Martín P., Ciorraga M. (2022). SoxD genes are required for adult neural stem cell activation. Cell Rep..

[23] Yang Z., Wang K.K.W. (2015). Glial fibrillary acidic protein: from intermediate filament assembly and gliosis to neurobiomarker. Trends Neurosci..

[24] Aoyama M., Kakita H., Kato S., Tomita M., Asai K. (2012). Region-specific expression of a water channel protein, aquaporin 4, on brain astrocytes. J. Neurosci. Res..

[25] Liddelow S.A., Guttenplan K.A., Clarke L.E., Bennett F.C., Bohlen C.J., Schirmer L., Bennett M.L., Münch A.E., Chung W.-S., Peterson T.C. (2017). Neurotoxic reactive astrocytes are induced by activated microglia. Nature.

[26] Choi D.-J., An J., Jou I., Park S.M., Joe E.-H. (2019). A Parkinson’s disease gene, DJ-1, regulates anti-inflammatory roles of astrocytes through prostaglandin D2 synthase expression. Neurobiol. Dis..

[27] Anderegg A., Poulin J.-F., Awatramani R. (2015). Molecular heterogeneity of midbrain dopaminergic neurons–Moving toward single cell resolution. FEBS Lett..

[28] Arenas E., Denham M., Villaescusa J.C. (2015). How to make a midbrain dopaminergic neuron. Development.

[29] Nair-Roberts R.G., Chatelain-Badie S.D., Benson E., White-Cooper H., Bolam J.P., Ungless M.A. (2008). Stereological estimates of dopaminergic, GABAergic and glutamatergic neurons in the ventral tegmental area, substantia nigra and retrorubral field in the rat. Neuroscience.

[30] Makrides N., Panayiotou E., Fanis P., Karaiskos C., Lapathitis G., Malas S. (2018). Sequential Role of SOXB2 Factors in GABAergic Neuron Specification of the Dorsal Midbrain. Front. Mol. Neurosci..

[31] Choi J.S., Ayupe A.C., Beckedorff F., Catanuto P., McCartan R., Levay K., Park K.K. (2023). Single-nucleus RNA sequencing of developing superior colliculus identifies neuronal diversity and candidate mediators of circuit assembly. Cell Rep..

[32] Tran H.-N., Nguyen Q.-H., Jeong Y. (2024). Dbx1 is a dorsal midbrain-specific determinant of GABAergic neuron fate and regulates differentiation of the dorsal midbrain into the inferior and superior colliculi. Front. Cell Dev. Biol..

[33] Düdükcü Ö., Raj D.D.A., van de Haar L.L., Grossouw L.M., Linders L.E., Garritsen O., Adolfs Y., van Kronenburg N.C.H., Broekhoven M.H., Kapteijns T.H.W. (2024). Molecular diversity and migration of GABAergic neurons in the developing ventral midbrain. iScience.

[34] Cooper M.A., Kobayashi K., Zhou R. (2009). Ephrin-A5 regulates the formation of the ascending midbrain dopaminergic pathways. Dev. Neurobiol..

[35] Spitzer N.C. (2015). Neurotransmitter Switching? No Surprise. Neuron.

[36] Tritsch N.X., Ding J.B., Sabatini B.L. (2012). Dopaminergic neurons inhibit striatal output through non-canonical release of GABA. Nature.

[37] Zhou F.-M., Liang Y., Salas R., Zhang L., De Biasi M., Dani J.A. (2005). Corelease of Dopamine and Serotonin from Striatal Dopamine Terminals. Neuron.

[38] Sulzer D., Joyce M.P., Lin L., Geldwert D., Haber S.N., Hattori T., Rayport S. (1998). Dopamine neurons make glutamatergic synapses in vitro. J. Neurosci..

[39] Siletti K., Hodge R., Mossi Albiach A., Lee K.W., Ding S.L., Hu L., Lönnerberg P., Bakken T., Casper T., Clark M. (2023). Transcriptomic diversity of cell types across the adult human brain. Science.

[40] Lin W., Metzakopian E., Mavromatakis Y.E., Gao N., Balaskas N., Sasaki H., Briscoe J., Whitsett J.A., Goulding M., Kaestner K.H. (2009). Foxa1 and Foxa2 function both upstream of and cooperatively with Lmx1a and Lmx1b in a feedforward loop promoting mesodiencephalic dopaminergic neuron development. Dev. Biol..

[41] Agoston Z., Li N., Haslinger A., Wizenmann A., Schulte D. (2012). Genetic and physical interaction of Meis2, Pax3 and Pax7 during dorsal midbrain development. BMC Dev. Biol..

[42] Chen K.H., Boettiger A.N., Moffitt J.R., Wang S., Zhuang X. (2015). Spatially resolved, highly multiplexed RNA profiling in single cells. Science.

[43] Moffitt J.R., Hao J., Wang G., Chen K.H., Babcock H.P., Zhuang X. (2016). High-throughput single-cell gene-expression profiling with multiplexed error-robust fluorescence in situ hybridization. Proc. Natl. Acad. Sci. USA.

[44] Redwine J.M., Evans C.F. (2002). Markers of central nervous system glia and neurons in vivo during normal and pathological conditions. Curr. Top. Microbiol. Immunol..

[45] Ladran I., Tran N., Topol A., Brennand K.J. (2013). Neural stem and progenitor cells in health and disease. Wiley Interdiscip. Rev. Syst. Biol. Med..

[46] Paşca A.M., Park J.Y., Shin H.W., Qi Q., Revah O., Krasnoff R., O’Hara R., Willsey A.J., Palmer T.D., Paşca S.P. (2019). Human 3D cellular model of hypoxic brain injury of prematurity. Nat. Med..

[47] Hubert C.G., Rivera M., Spangler L.C., Wu Q., Mack S.C., Prager B.C., Couce M., McLendon R.E., Sloan A.E., Rich J.N. (2016). A three-dimensional organoid culture system derived from human glioblastomas recapitulates the hypoxic gradients and cancer stem cell heterogeneity of tumors found in vivo. Cancer Res..

[48] Andrews M.G., Kriegstein A.R. (2022). Challenges of Organoid Research. Annu. Rev. Neurosci..

[49] Jacob F., Salinas R.D., Zhang D.Y., Nguyen P.T.T., Schnoll J.G., Wong S.Z.H., Thokala R., Sheikh S., Saxena D., Prokop S. (2020). A Patient-Derived Glioblastoma Organoid Model and Biobank Recapitulates Inter- and Intra-tumoral Heterogeneity. Cell.

[50] Kouli A., Torsney K.M., Kuan W.-L. (2018). Parkinson’s Disease: Etiology, Neuropathology, and Pathogenesis. Parkinsons Dis..

[51] Nalls M.A., Blauwendraat C., Vallerga C.L., Heilbron K., Bandres-Ciga S., Chang D., Tan M., Kia D.A., Noyce A.J., Xue A. (2019). Identification of novel risk loci, causal insights, and heritable risk for Parkinson’s disease: a meta-genome wide association study. Lancet Neurol..

[52] Foo J.N., Chew E.G.Y., Chung S.J., Peng R., Blauwendraat C., Nalls M.A., Mok K.Y., Satake W., Toda T., Chao Y. (2020). Identification of Risk Loci for Parkinson Disease in Asians and Comparison of Risk Between Asians and Europeans: A Genome-Wide Association Study. JAMA Neurol..

[53] Wang Q., Wang M., Choi I., Sarrafha L., Liang M., Ho L., Farrell K., Beaumont K.G., Sebra R., De Sanctis C. (2024). Molecular profiling of human substantia nigra identifies diverse neuron types associated with vulnerability in Parkinson’s disease.

[54] Pereira Luppi M., Azcorra M., Caronia-Brown G., Poulin J.-F., Gaertner Z., Gatica S., Moreno-Ramos O.A., Nouri N., Dubois M., Ma Y.C. (2021). Sox6 expression distinguishes dorsally and ventrally biased dopamine neurons in the substantia nigra with distinctive properties and embryonic origins. Cell Rep..

[55] Poulin J.-F., Zou J., Drouin-Ouellet J., Kim K.-Y.A., Cicchetti F., Awatramani R.B. (2014). Defining midbrain dopaminergic neuron diversity by single-cell gene expression profiling. Cell Rep..

[56] Buck S.A., Quincy Erickson-Oberg M., Logan R.W., Freyberg Z. (2022). Relevance of interactions between dopamine and glutamate neurotransmission in schizophrenia. Mol. Psychiatry.

[57] Casanovas S., Schlichtholz L., Mühlbauer S., Dewi S., Schüle M., Strand D., Strand S., Zografidou L., Winter J. (2020). Rbfox1 Is Expressed in the Mouse Brain in the Form of Multiple Transcript Variants and Contains Functional E Boxes in Its Alternative Promoters. Front. Mol. Neurosci..

[58] Kang H.J., Kawasawa Y.I., Cheng F., Zhu Y., Xu X., Li M., Sousa A.M.M., Pletikos M., Meyer K.A., Sedmak G. (2011). Spatio-temporal transcriptome of the human brain. Nature.

[59] Andres-Mateos E., Perier C., Zhang L., Blanchard-Fillion B., Greco T.M., Thomas B., Ko H.S., Sasaki M., Ischiropoulos H., Przedborski S. (2007). DJ-1 gene deletion reveals that DJ-1 is an atypical peroxiredoxin-like peroxidase. Proc. Natl. Acad. Sci. USA.

[60] van der Brug M.P., Blackinton J., Chandran J., Hao L.-Y., Lal A., Mazan-Mamczarz K., Martindale J., Xie C., Ahmad R., Thomas K.J. (2008). RNA binding activity of the recessive parkinsonism protein DJ-1 supports involvement in multiple cellular pathways. Proc. Natl. Acad. Sci. USA.

[61] Edson A.J., Hushagen H.A., Frøyset A.K., Elda I., Khan E.A., Di Stefano A., Fladmark K.E. (2019). Dysregulation in the Brain Protein Profile of Zebrafish Lacking the Parkinson’s Disease-Related Protein DJ-1. Mol. Neurobiol..

[62] Niere F., Namjoshi S., Song E., Dilly G.A., Schoenhard G., Zemelman B.V., Mechref Y., Raab-Graham K.F. (2016). Analysis of Proteins That Rapidly Change Upon Mechanistic/Mammalian Target of Rapamycin Complex 1 (mTORC1) Repression Identifies Parkinson Protein 7 (PARK7) as a Novel Protein Aberrantly Expressed in Tuberous Sclerosis Complex (TSC). Mol. Cell. Proteomics.

[63] Tang L., Xu N., Huang M., Yi W., Sang X., Shao M., Li Y., Hao Z.Z., Liu R., Shen Y. (2023). A primate nigrostriatal atlas of neuronal vulnerability and resilience in a model of Parkinson’s disease. Nat. Commun..

[64] Stojkovska I., Wani W.Y., Zunke F., Belur N.R., Pavlenko E.A., Mwenda N., Sharma K., Francelle L., Mazzulli J.R. (2022). Rescue of α-synuclein aggregation in Parkinson’s patient neurons by synergistic enhancement of ER proteostasis and protein trafficking. Neuron.

[65] Mazzulli J.R., Zunke F., Isacson O., Studer L., Krainc D. (2016). α-Synuclein–induced lysosomal dysfunction occurs through disruptions in protein trafficking in human midbrain synucleinopathy models. Proc. Natl. Acad. Sci. USA.

[66] Burbulla L.F., Song P., Mazzulli J.R., Zampese E., Wong Y.C., Jeon S., Santos D.P., Blanz J., Obermaier C.D., Strojny C. (2017). Dopamine oxidation mediates mitochondrial and lysosomal dysfunction in Parkinson’s disease. Science.

[67] Kumar S.T., Jagannath S., Francois C., Vanderstichele H., Stoops E., Lashuel H.A. (2020). How specific are the conformation-specific α-synuclein antibodies? Characterization and validation of 16 α-synuclein conformation-specific antibodies using well-characterized preparations of α-synuclein monomers, fibrils and oligomers with distinct struct. Neurobiol. Dis..

[68] Kovacs G.G., Wagner U., Dumont B., Pikkarainen M., Osman A.A., Streichenberger N., Leisser I., Verchère J., Baron T., Alafuzoff I. (2012). An antibody with high reactivity for disease-associated α-synuclein reveals extensive brain pathology. Acta Neuropathol..

[69] Lashuel H.A., Overk C.R., Oueslati A., Masliah E. (2013). The many faces of α-synuclein: from structure and toxicity to therapeutic target. Nat. Rev. Neurosci..

[70] Shan F.Y., Fung K.-M., Zieneldien T., Kim J., Cao C., Huang J.H. (2021). Examining the Toxicity of α-Synuclein in Neurodegenerative Disorders. Life.

[71] McCoy M.K., Cookson M.R. (2011). DJ-1 regulation of mitochondrial function and autophagy through oxidative stress. Autophagy.

[72] Chen R., Park H.-A., Mnatsakanyan N., Niu Y., Licznerski P., Wu J., Miranda P., Graham M., Tang J., Boon A.J.W. (2019). Parkinson’s disease protein DJ-1 regulates ATP synthase protein components to increase neuronal process outgrowth. Cell Death Dis..

[73] Almikhlafi M.A., Stauch K.L., Villeneuve L.M., Purnell P.R., Lamberty B.G., Fox H.S. (2020). Deletion of DJ-1 in rats affects protein abundance and mitochondrial function at the synapse. Sci. Rep..

[74] Lo C.H., Zeng J. (2023). Defective lysosomal acidification: a new prognostic marker and therapeutic target for neurodegenerative diseases. Transl. Neurodegener..

[75] Mahul-Mellier A.-L., Burtscher J., Maharjan N., Weerens L., Croisier M., Kuttler F., Leleu M., Knott G.W., Lashuel H.A. (2020). The process of Lewy body formation, rather than simply α-synuclein fibrillization, is one of the major drivers of neurodegeneration. Proc. Natl. Acad. Sci. USA.

[76] Surmeier D.J., Obeso J.A., Halliday G.M. (2017). Selective neuronal vulnerability in Parkinson disease. Nat. Rev. Neurosci..

[77] Coukos R., Krainc D. (2024). Key genes and convergent pathogenic mechanisms in Parkinson disease. Nat. Rev. Neurosci..

[78] Morrone Parfitt G., Coccia E., Goldman C., Whitney K., Reyes R., Sarrafha L., Nam K.H., Sohail S., Jones D.R., Crary J.F. (2024). Disruption of lysosomal proteolysis in astrocytes facilitates midbrain organoid proteostasis failure in an early-onset Parkinson’s disease model. Nat. Commun..

[79] Booth H.D.E., Hirst W.D., Wade-Martins R. (2017). The Role of Astrocyte Dysfunction in Parkinson’s Disease Pathogenesis. Trends Neurosci..

[80] Kyung J.W., Kim J.-M., Lee W., Ha T.-Y., Cha S.-H., Chung K.-H., Choi D.-J., Jou I., Song W.K., Joe E.-H. (2018). DJ-1 deficiency impairs synaptic vesicle endocytosis and reavailability at nerve terminals. Proc. Natl. Acad. Sci. USA.

[81] Camp J.G., Badsha F., Florio M., Kanton S., Gerber T., Wilsch-Bräuninger M., Lewitus E., Sykes A., Hevers W., Lancaster M. (2015). Human cerebral organoids recapitulate gene expression programs of fetal neocortex development. Proc. Natl. Acad. Sci. USA.

[82] Bhaduri A., Andrews M.G., Mancia Leon W., Jung D., Shin D., Allen D., Jung D., Schmunk G., Haeussler M., Salma J. (2020). Cell stress in cortical organoids impairs molecular subtype specification. Nature.

[83] Revah O., Gore F., Kelley K.W., Andersen J., Sakai N., Chen X., Li M.Y., Birey F., Yang X., Saw N.L. (2022). Maturation and circuit integration of transplanted human cortical organoids. Nature.

[84] Miura Y., Li M.-Y., Revah O., Yoon S.-J., Narazaki G., Pașca S.P. (2022). Engineering brain assembloids to interrogate human neural circuits. Nat. Protoc..

[85] Pachitariu M., Stringer C. (2022). Cellpose 2.0: how to train your own model. Nat. Methods.

[86] Sullivan D.K., Min K.H., Hjörleifsson K.E., Luebbert L., Holley G., Moses L., Gustafsson J., Bray N.L., Pimentel H., Booeshaghi A.S. (2024). kallisto, bustools and kb-python for quantifying bulk, single-cell and single-nucleus RNA-seq. Nat. Protoc..

[87] Bergen V., Lange M., Peidli S., Wolf F.A., Theis F.J. (2020). Generalizing RNA velocity to transient cell states through dynamical modeling. Nat. Biotechnol..

[88] Stuart T., Butler A., Hoffman P., Hafemeister C., Papalexi E., Mauck W.M., Hao Y., Stoeckius M., Smibert P., Satija R. (2019). Comprehensive Integration of Single-Cell Data. Cell.

[89] Andreatta M., Carmona S.J. (2021). UCell: Robust and scalable single-cell gene signature scoring. Comput. Struct. Biotechnol. J..

[90] Love M.I., Huber W., Anders S. (2014). Moderated estimation of fold change and dispersion for RNA-seq data with DESeq2. Genome Biol..

[91] Kolde R. (2018). pheatmap: Pretty Heatmaps. https://github.com/raivokolde/pheatmap/issues.

[92] Venables W.N., Ripley B.D. (2002).

[93] Wu T., Hu E., Xu S., Chen M., Guo P., Dai Z., Feng T., Zhou L., Tang W., Zhan L. (2021). clusterProfiler 4.0: A universal enrichment tool for interpreting omics data. Innovation.

[94] Strimmer K. (2008). A unified approach to false discovery rate estimation. BMC Bioinf..

[95] Csárdi G., Nepusz T., Traag V., Horvát S., Zanini F., Noom D., Müller K. (2025). igraph: Network Analysis and Visualization in R.

[96] Gu Z., Eils R., Schlesner M. (2016). Complex heatmaps reveal patterns and correlations in multidimensional genomic data. Bioinformatics (Oxford, England).

[97] Korsunsky I., Millard N., Fan J., Slowikowski K., Zhang F., Wei K., Baglaenko Y., Brenner M., Loh P.R., Raychaudhuri S. (2019). Fast, sensitive and accurate integration of single-cell data with Harmony. Nat. Methods.

[98] Soneson C., Love M.I., Robinson M.D. (2015). Differential analyses for RNA-seq: transcript-level estimates improve gene-level inferences. F1000Res..

[99] Miyoshi G., Fishell G. (2012). Dynamic FoxG1 expression coordinates the integration of multipolar pyramidal neuron precursors into the cortical plate. Neuron.

[100] Cargnin F., Kwon J.-S., Katzman S., Chen B., Lee J.W., Lee S.-K. (2018). FOXG1 Orchestrates Neocortical Organization and Cortico-Cortical Connections. Neuron.

[101] Muzio L., Di Benedetto B., Stoykova A., Boncinelli E., Gruss P., Mallamaci A. (2002). Emx2 and Pax6 control regionalization of the pre-neuronogenic cortical primordium. Cereb. Cortex.

[102] Quinn J.C., Molinek M., Martynoga B.S., Zaki P.A., Faedo A., Bulfone A., Hevner R.F., West J.D., Price D.J. (2007). Pax6 controls cerebral cortical cell number by regulating exit from the cell cycle and specifies cortical cell identity by a cell autonomous mechanism. Dev. Biol..

[103] Tiklová K., Nolbrant S., Fiorenzano A., Björklund Å.K., Sharma Y., Heuer A., Gillberg L., Hoban D.B., Cardoso T., Adler A.F. (2020). Single cell transcriptomics identifies stem cell-derived graft composition in a model of Parkinson’s disease. Nat. Commun..

[104] Herbert J., Cavallaro T., Dwork A.J. (1990). A marker for primary choroid plexus neoplasms. Am. J. Pathol..

[105] Johansson P.A., Dziegielewska K.M., Ek C.J., Habgood M.D., Møllgård K., Potter A., Schuliga M., Saunders N.R. (2005). Aquaporin-1 in the choroid plexuses of developing mammalian brain. Cell Tissue Res..

[106] Oberwinkler J., Lis A., Giehl K.M., Flockerzi V., Philipp S.E. (2005). Alternative Splicing Switches the Divalent Cation Selectivity of TRPM3 Channels. J. Biol. Chem..

[107] Hafemeister C., Satija R. (2019). Normalization and variance stabilization of single-cell RNA-seq data using regularized negative binomial regression. Genome Biol..

[108] Choudhary S., Satija R. (2022). Comparison and evaluation of statistical error models for scRNA-seq. Genome Biol..

[109] Cleveland W.S. (1993).

