# Supplementary material for: Single-cell transcriptomics revealed molecular vulnerability in a human midbrain-like organoid model of Parkinson’s disease
Source: iScience. 2026 Jan 10;29(2):114674. doi: 10.1016/j.isci.2026.114674 (PMC12918236; doi:10.1016/j.isci.2026.114674)
Supplement: Document S1. Figures S1–S6 and Tables S1–S4 [file mmc1.pdf]

iScience, Volume *29*

Supplemental information

Single-cell transcriptomics revealed molecular vulnerability in a human midbrain-like organoid model of Parkinson's disease

Jessica Jiaxin Xie, Matas Vitkauskas, Quyen Do, Tzuen Yih Saw, Alfred Xuyang Sun, Lin Yang, Tuck Wah Soong, Kah-Leong Lim, Eng-King Tan, Huck-Hui Ng, and Jinyue Liu

Fig S1

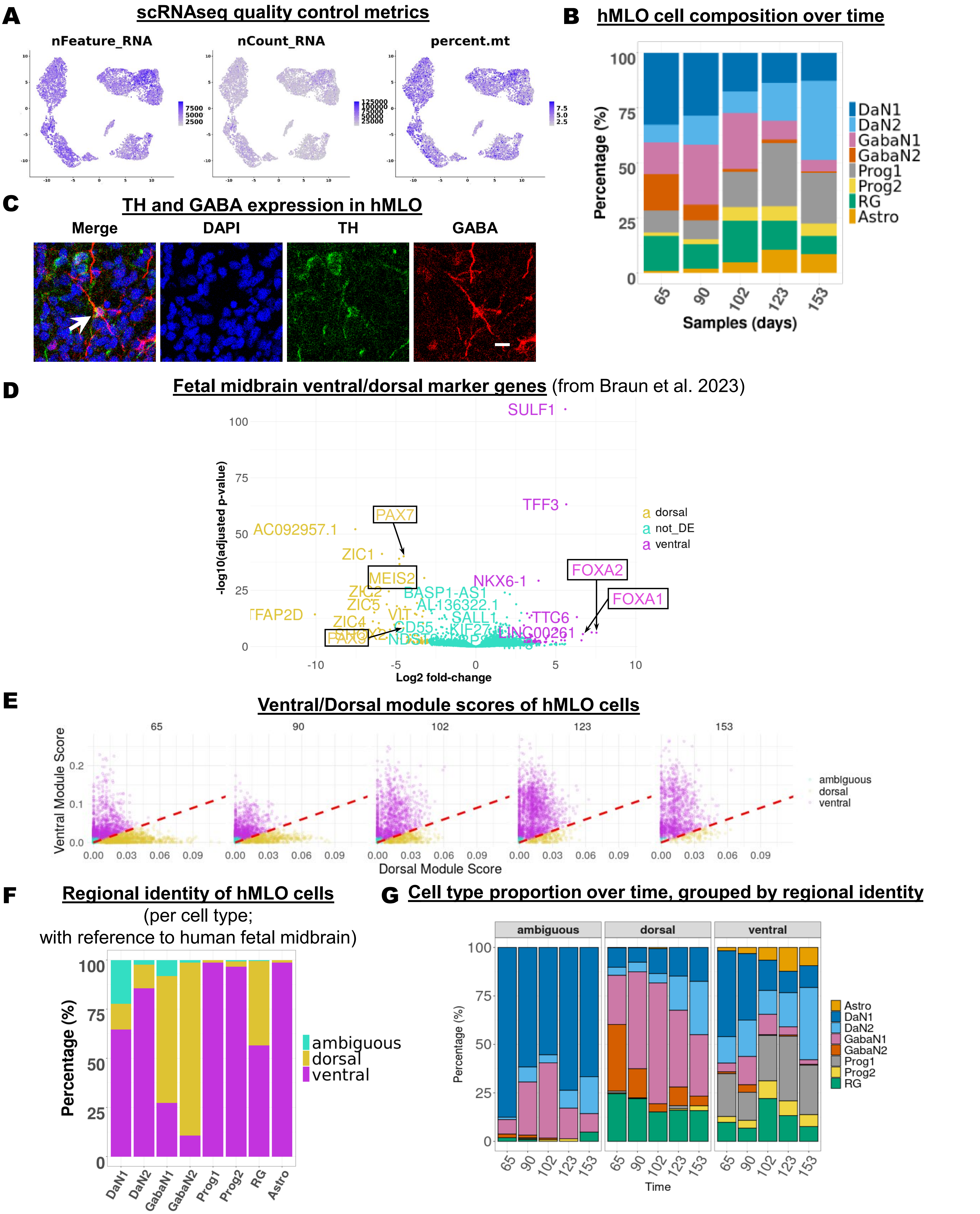
A
scRNAseq quality control metrics
nFeature_RNA
nCount_RNA
percent.mt
B
hMLO cell composition over time
Percentage (%)
Samples (days)
65
90
102
123
153
DaN1
DaN2
GabaN1
GabaN2
Prog1
Prog2
RG
Astro
C
TH and GABA expression in hMLO
Merge
DAPI
TH
GABA
D
Fetal midbrain ventral/dorsal marker genes (from Braun et al. 2023)
-log10(adjusted p-value)
Log2 fold-change
SULF1
TFF3
PAX7
AC092957.1
ZIC1
MEIS2
NKX6-1
FOXA2
FOXA1
BASP1-AS1
AL136322.1
SALL1
TTC6
TFAP2D
VIT
ZIC4
CD55
KIF27
LINC00261
PAX3
dorsal
not_DE
ventral
E
Ventral/Dorsal module scores of hMLO cells
Ventral Module Score
Dorsal Module Score
ambiguous
dorsal
ventral
F
Regional identity of hMLO cells
(per cell type;
with reference to human fetal midbrain)
Percentage (%)
ambiguous
dorsal
ventral
DaN1
DaN2
GabaN1
GabaN2
Prog1
Prog2
RG
Astro
G
Cell type proportion over time, grouped by regional identity
ambiguous
dorsal
ventral
Percentage (%)
Time
Astro
DaN1
DaN2
GabaN1
GabaN2
Prog1
Prog2
RG

FIG S2

A

136 MERFISH panel genes

ACHE, AJAP1, ANK1, AQP4, ARRDC3, ARSA, ASIC4, ATP13A2, ATP8A2, BDNF, BTG1, CACNA1B, CALB1, CHD5, CHGB, CHST1, CNGB1, CREG2, CSMD2, CST3, CTSC, DCX, DDC, DNAJA4, DNAJC15, DNAJC6, DNMT3B, DRD2, E2F8, EGLN3, ENO2, FAR2, FECH, FGF14, FN1, GAD1, GALNT10, GCK, GPI, GPR50, HEPACAM2, HMCN1, HMGCR, HMGCS1, HPCAL4, IFIH1, IGFBP3, IRAK1, ITPR2, KCNA1, KCNB2, KCNJ5, KCNJ6, KCNJ9, L1CAM, LAMB1, LMX1A, LMX1B, LOX, MAOA, MAOB, MAPT, MGAT4C, NDNF, NETO1, NEUROD1, NEUROD2, NHLH1, NR4A2, NSG1, NTM, NTRK3, OPRK1, OTX2, P2RX3, P4HA1, PAM, PCSK2, PDE2A, PDGFB, PDYN, PFKL, PFKP, PGK1, PGM1, PLAT, PLXNC1, PPP1R3B, PPP2R5B, PRDM13, PRDM8, PROKR1, RAB3B, RCAN2, RET, RYR2, SCG2, SCN1A, SCN2A, SERPINE1, SLC17A6, SLC18A1, SLC1A3, SLC6A17, SNAP25, SNCA, SOD2, SOX6, SPINK5, SQLE, SSTR2, STC1, STC2, STMN2, STMN4, STYK1, SV2B, SYT1, SYT4, SYT5, SYTL5, TF, TGFA, TGFB3, THY1, TMCC3, TMEFF2, TPH1, TXNIP, UHRF1, VEGFA, VSNL1, ZCCHC12, ZIC1, ZIC5, ZNF667

B

Correlation of transcript count with FPKM

(one MERFISH experiment)

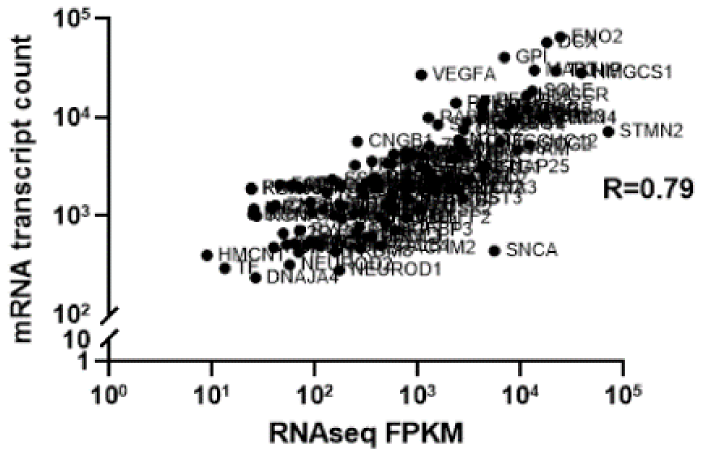

C

Correlation values of transcript count vs FPKM

(all MERFISH datasets)

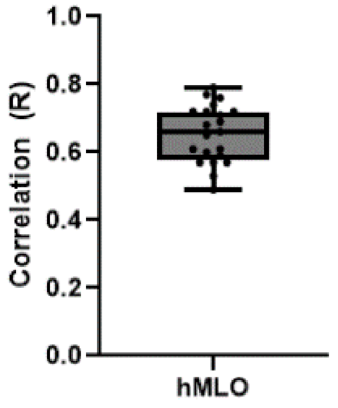

D

Tissue map of three WT hMLOs (day 60)

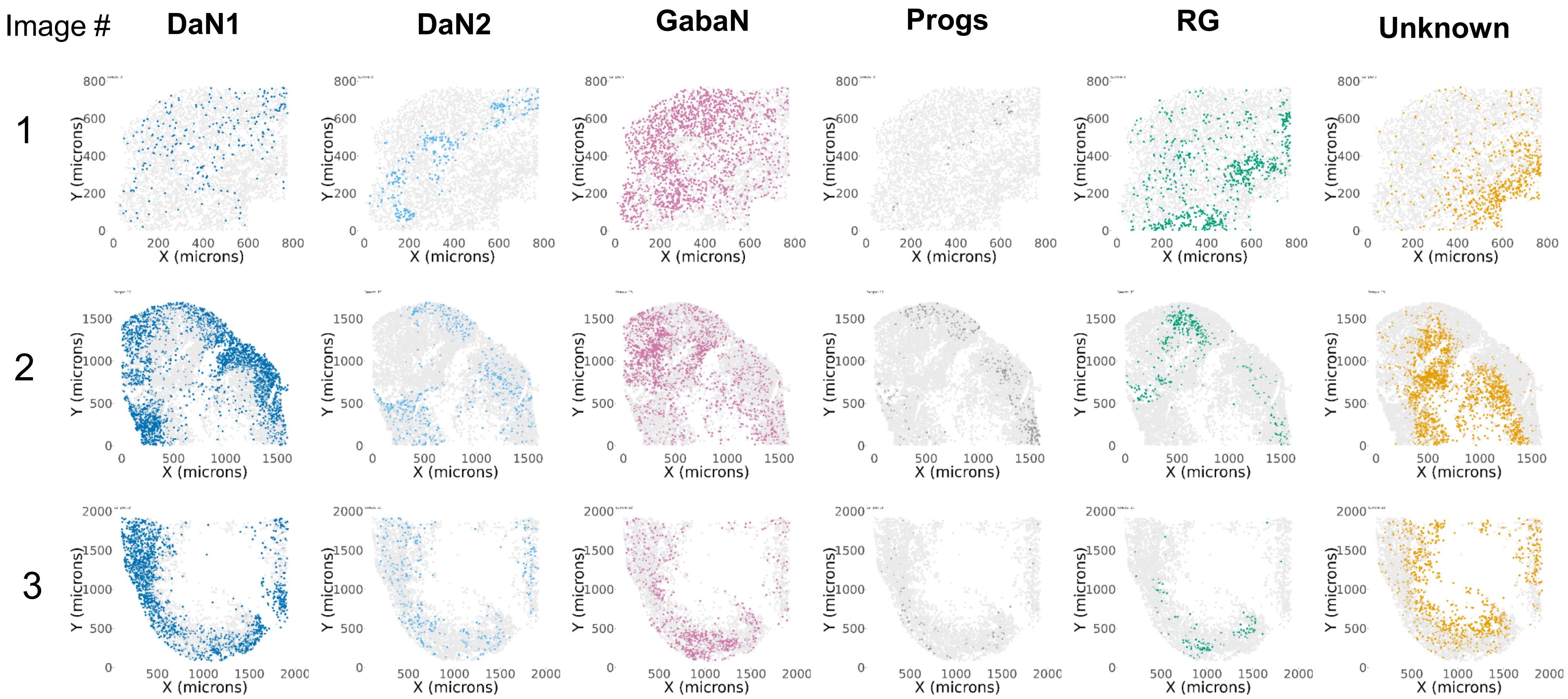

Fig S3

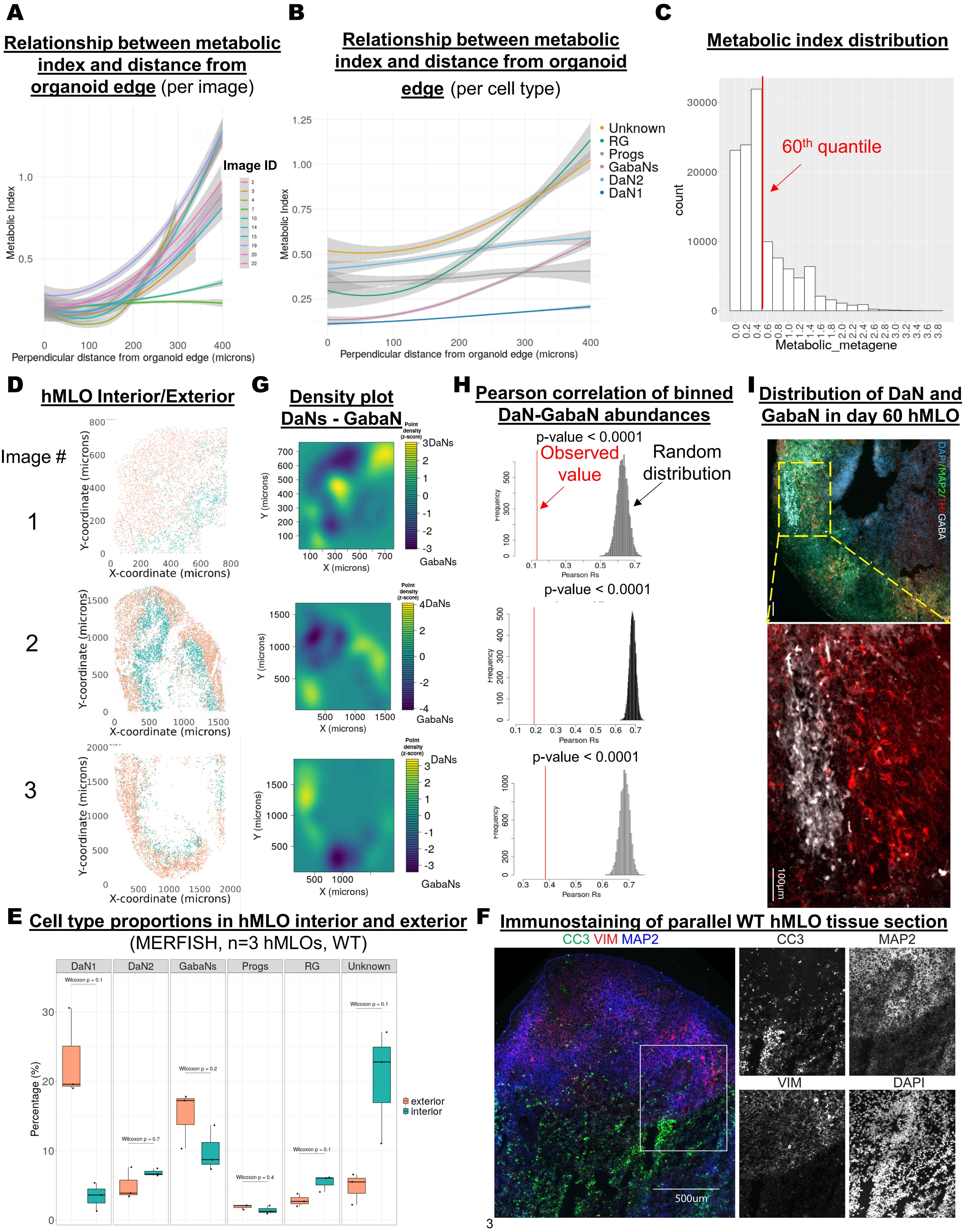
A
Relationship between metabolic index and distance from organoid edge (per image)
Metabolic Index
Perpendicular distance from organoid edge (microns)
Image ID
B
Relationship between metabolic index and distance from organoid edge (per cell type)
Unknown
RG
Progs
GabaNs
DaN2
DaN1
Metabolic Index
Perpendicular distance from organoid edge (microns)
C
Metabolic index distribution
60th quantile
count
Metabolic_metagene
D
hMLO Interior/Exterior
Image #
1
2
3
Y-coordinate (microns)
X-coordinate (microns)
G
Density plot DaNs - GabaN
Point density (z-score)
DaNs
GabaNs
Y (microns)
X (microns)
H
Pearson correlation of binned DaN-GabaN abundances
p-value < 0.0001
Observed value
Random distribution
Frequency
Pearson Rs
p-value < 0.0001
p-value < 0.0001
I
Distribution of DaN and GabaN in day 60 hMLO
DAPI/MAP2/TH/GABA
100um
E
Cell type proportions in hMLO interior and exterior (MERFISH, n=3 hMLOs, WT)
DaN1
DaN2
GabaNs
Progs
RG
Unknown
Wilcoxon p = 0.1
Wilcoxon p = 0.7
Wilcoxon p = 0.2
Wilcoxon p = 0.4
Wilcoxon p = 0.1
Wilcoxon p = 0.1
Percentage (%)
exterior
interior
F
Immunostaining of parallel WT hMLO tissue section
CC3 VIM MAP2
CC3
MAP2
VIM
DAPI
500um

FIG S4

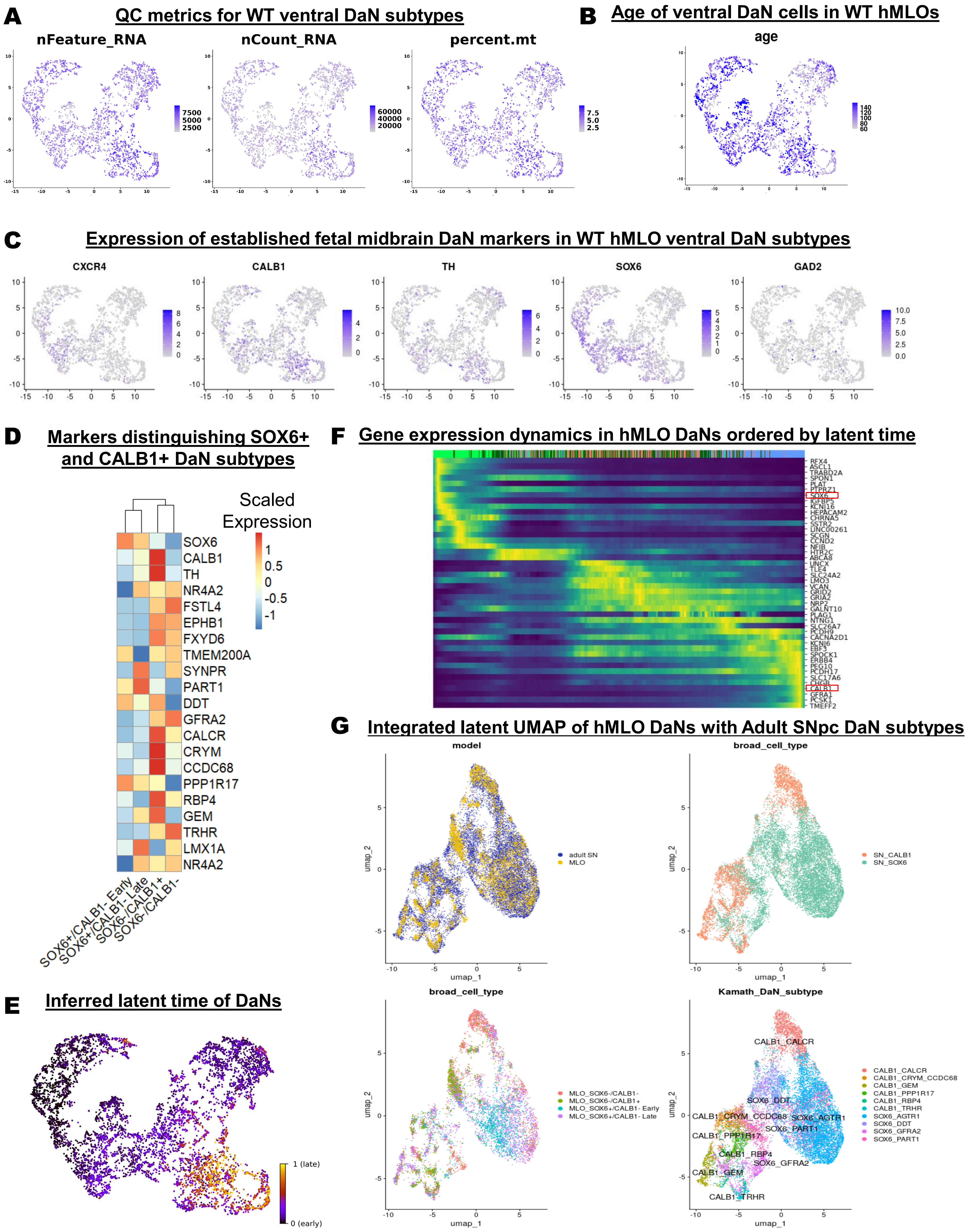
A
QC metrics for WT ventral DaN subtypes
nFeature_RNA
nCount_RNA
percent.mt
7500
5000
2500
60000
40000
20000
7.5
5.0
2.5
B
Age of ventral DaN cells in WT hMLOs
age
140
120
100
80
60
C
Expression of established fetal midbrain DaN markers in WT hMLO ventral DaN subtypes
CXCR4
CALB1
TH
SOX6
GAD2
D
Markers distinguishing SOX6+ and CALB1+ DaN subtypes
Scaled Expression
1
0.5
0
-0.5
-1
SOX6
CALB1
TH
NR4A2
FSTL4
EPHB1
FXYD6
TMEM200A
SYNPR
PART1
DDT
GFRA2
CALCR
CRYM
CCDC68
PPP1R17
RBP4
GEM
TRHR
LMX1A
NR4A2
SOX6+/CALB1- Early
SOX6+/CALB1- Late
SOX6-/CALB1+
SOX6-/CALB1-
E
Inferred latent time of DaNs
1 (late)
0 (early)
F
Gene expression dynamics in hMLO DaNs ordered by latent time
RFX4
ASCL1
TRABD2A
SPON1
PLAT
PTPRZ1
SOX6
IGFBP5
KCNJ16
HEPACAM2
CHRNA5
SSTR2
LINC00261
SCGN
CCND2
NFIB
HTR2C
ABCA8
UNCX
TLE4
SLC24A2
LMO3
VCAN
GRID2
GRIA2
NRP2
GALNT10
PLAG1
NTNG1
SLC26A7
PCDH9
CACNA2D1
KCNJ6
EBF3
SPOCK1
ERBB4
PEG10
PCDH17
SLC17A6
CHGB
CALB1
GFRA1
PCSK1
TMEFF2
G
Integrated latent UMAP of hMLO DaNs with Adult SNpc DaN subtypes
model
adult SN
MLO
broad_cell_type
SN_CALB1
SN_SOX6
broad_cell_type
MLO_SOX6-/CALB1-
MLO_SOX6-/CALB1+
MLO_SOX6+/CALB1- Early
MLO_SOX6+/CALB1- Late
Kamath_DaN_subtype
CALB1_CALCR
CALB1_CRYM_CCDC68
CALB1_GEM
CALB1_PPP1R17
CALB1_RBP4
CALB1_TRHR
SOX6_AGTR1
SOX6_DDT
SOX6_GFRA2
SOX6_PART1
umap_1
umap_2

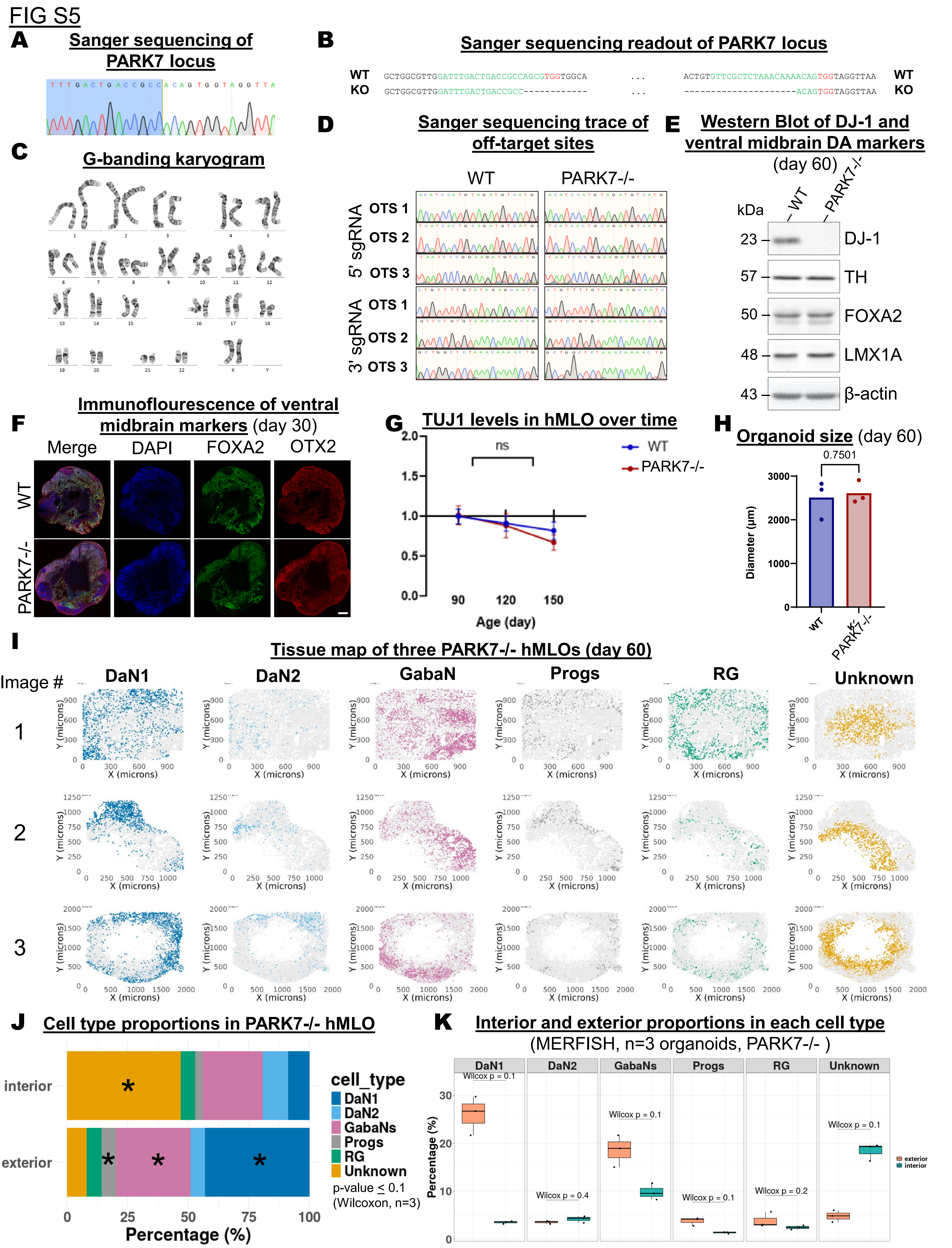

FIG S5
A
Sanger sequencing of PARK7 locus
B
Sanger sequencing readout of PARK7 locus
WT GCTGGCGTTGGATTTGACTGACCGCCAGCGTGGTGGCA ... ACTGTGTTCGCTCTAAACAAAACAGTGGTAGGTTAA WT
KO GCTGGCGTTGGATTTGACTGACCGCC------------ ... ---------------------ACAGTGGTAGGTTAA KO
C
G-banding karyogram
D
Sanger sequencing trace of off-target sites
WT
PARK7-/-
5' sgRNA
OTS 1
OTS 2
OTS 3
3' sgRNA
OTS 1
OTS 2
OTS 3
E
Western Blot of DJ-1 and ventral midbrain DA markers
(day 60)
kDa
WT
PARK7-/-
23 DJ-1
57 TH
50 FOXA2
48 LMX1A
43 β-actin
F
Immunoflourescence of ventral midbrain markers (day 30)
Merge
DAPI
FOXA2
OTX2
WT
PARK7-/-
G
TUJ1 levels in hMLO over time
ns
WT
PARK7-/-
Age (day)
90
120
150
H
Organoid size (day 60)
0.7501
Diameter (μm)
WT
PARK7-/-
I
Tissue map of three PARK7-/- hMLOs (day 60)
Image #
DaN1
DaN2
GabaN
Progs
RG
Unknown
1
2
3
Y (microns)
X (microns)
J
Cell type proportions in PARK7-/- hMLO
interior
exterior
cell_type
DaN1
DaN2
GabaNs
Progs
RG
Unknown
p-value ≤ 0.1
(Wilcoxon, n=3)
Percentage (%)
K
Interior and exterior proportions in each cell type
(MERFISH, n=3 organoids, PARK7-/-)
DaN1
DaN2
GabaNs
Progs
RG
Unknown
Wilcox p = 0.1
Wilcox p = 0.4
Wilcox p = 0.1
Wilcox p = 0.1
Wilcox p = 0.2
Wilcox p = 0.1
Percentage (%)
exterior
interior

Fig S6

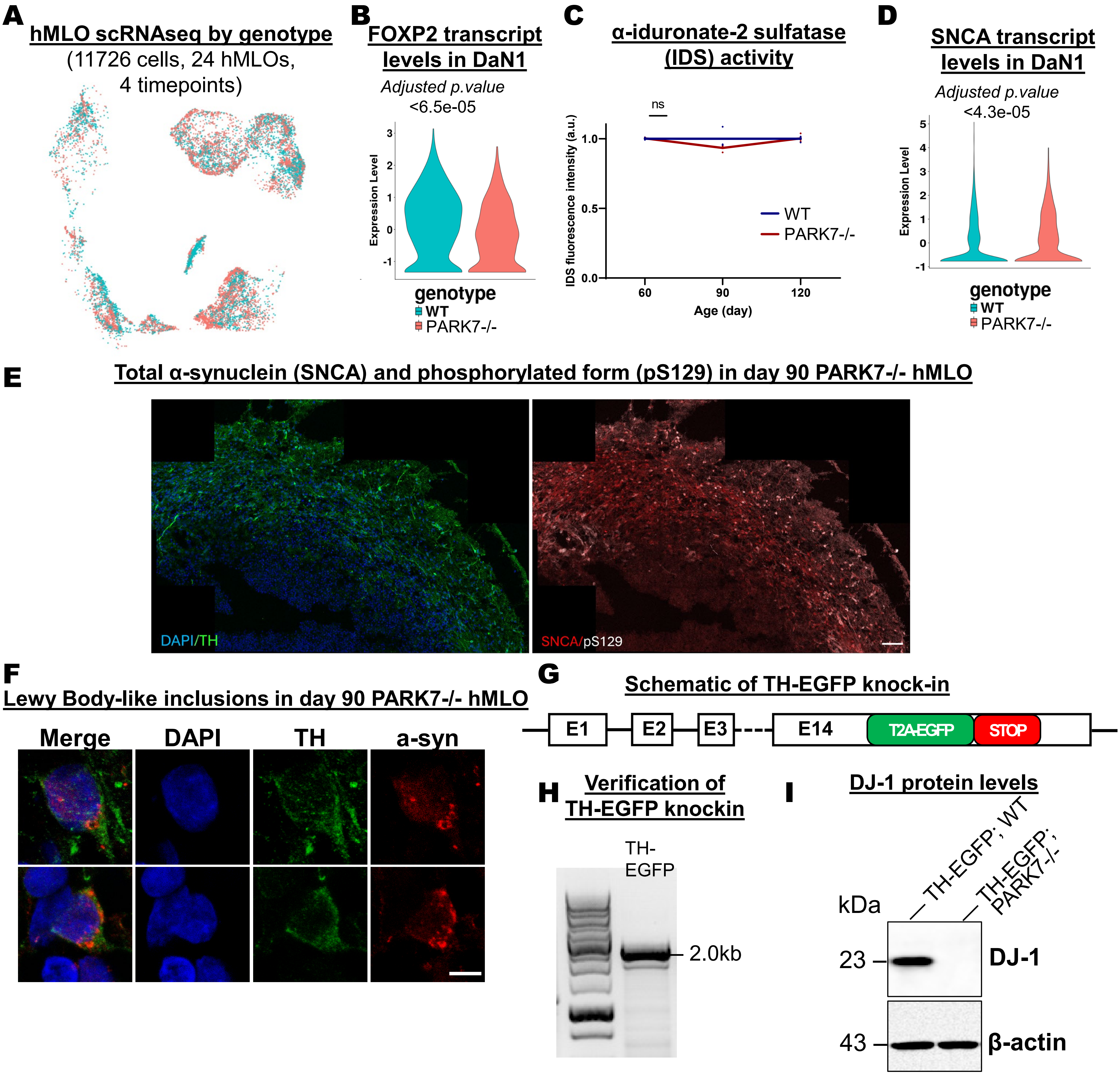
A
hMLO scRNAseq by genotype
(11726 cells, 24 hMLOs,
4 timepoints)
B
FOXP2 transcript
levels in DaN1
Adjusted p.value
<6.5e-05
Expression Level
genotype
WT
PARK7-/-
C
α-iduronate-2 sulfatase
(IDS) activity
ns
IDS fluorescence intensity (a.u.)
WT
PARK7-/-
Age (day)
D
SNCA transcript
levels in DaN1
Adjusted p.value
<4.3e-05
Expression Level
genotype
WT
PARK7-/-
E
Total α-synuclein (SNCA) and phosphorylated form (pS129) in day 90 PARK7-/- hMLO
DAPI/TH
SNCA/pS129
F
Lewy Body-like inclusions in day 90 PARK7-/- hMLO
Merge
DAPI
TH
a-syn
G
Schematic of TH-EGFP knock-in
E1
E2
E3
E14
T2A-EGFP
STOP
H
Verification of
TH-EGFP knockin
TH-
EGFP
2.0kb
I
DJ-1 protein levels
TH-EGFP; WT
TH-EGFP; PARK7-/-
kDa
23
43
DJ-1
β-actin

SUPPLEMENTARY DATA

Fig S1: Single-cell characterization of hMLOs and published human midbrain atlases.

A. UMAP scatterplot of 29053 scRNAseq transcriptomic profiles of day 60–153 wildtype (WT) hMLO cells coloured by quality control metrics. nFeature_RNA: number of genes per cell; nCount_RNA: number of molecules per cell; percent.mt: percentage mitochondrial genome per cell.
B. Split bar chart showing hMLO cell composition over time.
C. Immunostaining showing TH and GABA co-expression in day 60 hMLO. Scale bar: 10 µm.
D. Volcano plot showing fold-change and adjusted p-values of genes differentially expressed in ventral (green) and dorsal midbrain (red). Differential expression was defined as baseMean expression > 200, p-value < 0.05 and absolute log-2-fold-change > 3.
E. Scatter plot showing ventral and dorsal module scores of hMLO cells across different time points. Each dot is a cell.
F. Stacked barplot indicating proportions of each regional identity per hMLO cell type.
G. Stacked barplot of cell type proportion over time, grouped by regional identity. Region labels are with reference to human fetal midbrain.

Fig S2: Quality checks of hMLO MERFISH datasets.

A. List of 136 genes in the MERFISH panel.
B. Correlation of transcript count and FPKM expression in hMLOs, as measured by MERFISH and bulk RNA sequencing respectively.
C. Bar graph showing correlation of transcript count with FPKM for all MERFISH datasets performed (R=0.65 ± 0.085).
D. Tissue maps of cell centroids in three different WT hMLOs. Each colour corresponds to a different cell type identity.

Fig S3. Characterization of metabolic index, DaN/GabaN distributions and cell type proportions in hMLO MERFISH.

A. Line plot of Metabolic index relationship to distance from organoid edge (per image).
B. Line plot of Metabolic index relationship to distance from organoid edge (per cell type).
C. Histogram of metabolic index distribution. Red line indicates the cutoff for defining hMLO interior and exterior (60th quantile).
D. Tissue map of three WT hMLOs showing cells marked as interior or exterior based on their binarized metabolic index.
E. Boxplot showing percentage of interior and exterior cells per image for each cell type in WT hMLO (Wilcoxon ranked-sum test). Each dot is a hMLO. Center line is median and whiskers are 1.5x interquartile range.
F. Immunofluorescence images of a parallel day 60 WT hMLO section stained for proliferation marker VIM, necrotic marker CC3 and neuronal marker MAP2. Scale: 500µm.
G. Contour plot of point density difference between DaNs and GabaNs in WT hMLOs.
H. Pearson correlation values of binned cell centroids of DaNs versus those of GabaNs (in red) for three images. Corresponding histogram of 10000 Pearson correlation values obtained from permuting binned cell centroids of DaNs and GabaNs are marked "random distribution". p-value < 0.0001, one-tailed permutation test.
I. Immunofluorescence for MAP2, TH and GABA in day 60 hMLO. TH marks DaNs, GABA marks GabaNs and MAP2 marks all neurons. Scale bar=100µm.

Fig S4: Characterization of SOX6-CALB1 transcriptional axis within hMLO DaN subtypes.

A. UMAP scatterplot of hMLO ventral DaN subtypes coloured by quality control metrics: nFeature_RNA: number of genes per cell; nCount_RNA: number of molecules per cell; percent.mt: percentage mitochondrial genome per cell.
B. scRNAseq UMAP scatterplot of hMLO ventral DaNs by age.
C. UMAP scatterplot colored by expression of fetal midbrain DaN subtype markers from Braun et al 2023[34].
D. Heatmap showing scaled expression in hMLOs of genes that are known to be differentially expressed between SOX6+ and SOX6- DaN subtypes[14,54].
E. UMAP of inferred latent time of DaNs.
F. Gene expression dynamics in hMLO DaNs ordered by latent time.
G. UMAP embeddings of shared (integrated) latent space between MLO and adult substantia nigra pars compacta (SNpc) cells from Kamath et al. 2022[14], coloured by model system, broad adult SNpc subtypes, MLO subtypes or Kamath subtypes.

Fig S5: **Characterization of PARK7-/- hMLOs.**

A. Sanger sequencing trace chromatogram of PARK7 locus of the PARK7-/- hESC line.
B. Readout of trace chromatogram of the PARK7 locus of the PARK7-/- hESC line.
C. G-banding karyogram of the PARK7-/- hESC line.
D. Sanger sequencing trace chromatograms of top off-target sites of the isogenic WT and PARK7-/- hESC lines.
E. Western blots showing protein expression of DJ-1 and ventral midbrain DA markers in day 60 hMLO.
F. Immunofluorescence of ventral midbrain markers in day 30 hMLO. Scale bar: 200µm
G. Quantification of TUJ1 levels in WT and PARK7-/- hMLOs over time (n=3 independent batches at each timepoint).
H. Barplot showing quantification of day 60 hMLO size (n=3 independent batches).
I. Tissue maps of cell centroids corresponding to different cell type identities in PARK7-/- hMLOs.
J. Barplot showing mean cell type proportions across all PARK7-/- samples in hMLO interior or exterior. Asterisks indicate cell types for which the average percentages of cells that are in the hMLO interior were significantly different from that in the exterior ($p \leq 0.1$, Wilcoxon ranked sum test).
K. Boxplot showing percentage of interior and exterior cells per sample for each cell type in PARK7-/- hMLO (Wilcoxon ranked-sum test). Center line is median and whiskers are 1.5x interquartile range.

Fig S6: Cell type proportions, gene expression and phenotypes in PARK7-/- hMLOs, and verification of TH-EGFP knock-in.

A. UMAP scatterplot of scRNAseq transcriptomic profiles of H9 hESC-derived hMLO cells (n=24 hMLOs from 4 timepoints), coloured by genotype.
B. Violin plot of scaled FOXP2 expression per genotype in DaN1 (z-scores). Data plotted were downsampled to equal number of cells per genotype.
C. Enzyme activity of IDS in hMLO at indicated timepoints (d60 n=4; d90 n=3; d120 n=5 independent batches each for WT and PARK7-/-).
D. Violin plot of scaled SNCA expression per genotype in DaN1 (z-scores). Data plotted were downsampled to equal number of cells per genotype.

E. TH (green), Total α-synuclein (SNCA, red) and phosphorylated form (pS129, white) in day 90 PARK7-/- hMLO.
F. Lewy Body-like inclusions identified by immunofluorescence of TH and α-Synuclein in day 90 PARK7-/- hMLO. Scale bar: 10μm.
G. Schematic showing strategy for generation of TH-EGFP cell line in H9 background.
H. Gel electrophoresis blot validating insertion of T2A-EGFP construct.
I. Western blot validating absence of DJ-1 protein as a result of PARK7 knockout in TH-EGFP hESC line.

Supplementary table 1: scRNAseq metadata (WT samples, n = 3 per S/N)

S/N	Age (days of differentiation)	Genotype	Organoid differentiation batch #	Single-cell dissociation batch #	Sequencing batch #	Library prep method	# of cells
1	60	WT	1	1	1	Manual	2821
2	105	WT	2	2	1	Manual	4280
3	99	WT	3	2	1	Manual	4120
4	126	WT	2	3	1	Manual	3354
5	120	WT	3	3	1	Manual	3268
6	126	WT	4	4	1	Automated	2590
7	156	WT	5	5	1	Manual	3189
8	70	WT	6	6	2	Manual	2600
9	90	WT	7	7	2	Manual	2080
10	120	WT	7	8	2	Manual	162
11	150	WT	7	9	2	Manual	589

Supplementary table 2: MERFISH metadata

S/N	Sample ID	Age (days of differentiation)	Genotype	Organoid #	Organoid differentiation batch #	Cryosection #	Imaging batch #	# of cells
1	2	60	WT	1	1	1	1	13929
2	3	60	WT	1	1	2	2	7745
3	4	60	WT	1	1	2	2	8232
4	7	60	WT	1	1	3	3	5031
5	10	60	WT	1	1	4	4	6743
6	14	60	WT	2	2	5	5	3368
7	15	60	WT	2	2	6	6	4311
8	19	60	WT	3	3	7	7	3289
9	20	60	WT	3	3	7	7	3166
10	22	60	WT	3	3	8	8	6426

Supplementary table 3: sPD genes above baseline expression level (Sequencing-depth corrected mean expression > 1, total 93 sPD genes)

Cell type	# of total genes above baseline	# sPD genes above baseline	Gene names	# of sPD genes that are not expressed
GabaN2	1733	19	KRTCAP2, NUCKS1, SIPA1L2, MAP4K4, IP6K2, LCORL, CAMK2D, CLCN3, RIMS1,	5

			FYN, RPS12, FAM49B, DLG2, HIP1R, CHD9, ASXL3, MED13, DYRK1A, SV2C	
GabaN1	1801	18	KRTCAP2, NUCKS1, SIPA1L2, MAP4K4, IP6K2, LCORL, CAMK2D, PAM, RIMS1, FYN, RPS12, FAM49B, SH3GL2, DLG2, CHD9, ASXL3, DYRK1A, GALNT17	2
DaN2	1741	17	KRTCAP2, NUCKS1, MAP4K4, TMEM163, IP6K2, LCORL, CAMK2D, PAM, RIMS1, FYN, RPS12, FAM49B, DLG2, MIPOL1	0
DaN1	1991	18	KRTCAP2, NUCKS1, MAP4K4, TMEM163, IP6K2, LCORL, SNCA, CAMK2D, PAM, RIMS1, FYN, RPS12, FAM49B, DLG2	0
Astro	1692	15	PMVK, KRTCAP2, NUCKS1, SIPA1L2, TMEM163, SCARB2, CAMK2D, PAM, FYN, RPS12, CTSB, SH3GL2, DLG2, CHD9, GRN	3
Prog2	1557	14	KRTCAP2, NUCKS1, SIPA1L2, TMEM163, SCARB2, SNCA, CAMK2D, PAM, FYN, RPS12, CTSB, DLG2, CHD9, GRN	1
Prog1	1689	11	KRTCAP2, NUCKS1, TMEM163, CAMK2D, PAM, FYN, RPS12, CTSB, DLG2, CHD9, GRN,	2
RG	1648	9	KRTCAP2, NUCKS1, CAMK2D, FYN, RPS12, DLG2, SCAF11, CHD9, GRN	1

Supplementary table 4: scRNAseq metadata (KO samples, n = 3 per S/N)

S/N	Age (days of differentiation)	Genotype	Organoid differentiation batch #	Single-cell dissociation batch #	Sequencing batch #	Library prep method	# of cells
1	70	DJ-1 KO	6	6	2	Manual	3489
2	90	DJ-1 KO	7	7	2	Manual	1326
3	120	DJ-1 KO	7	8	2	Manual	190
4	150	DJ-1 KO	7	9	2	Manual	1290
